# Chlorquinaldol Alleviates Lung Fibrosis in Mice by Inhibiting Fibroblast Activation through Targeting Methionine Synthase Reductase

**DOI:** 10.1021/acscentsci.4c00798

**Published:** 2024-08-28

**Authors:** Xiangyu Yang, Geng Lin, Yitong Chen, Xueping Lei, Yitao Ou, Yuyun Yan, Ruiwen Wu, Jie Yang, Yiming Luo, Lixin Zhao, Xiuxiu Zhang, Zhongjin Yang, Aiping Qin, Ping Sun, Xi-Yong Yu, Wenhui Hu

**Affiliations:** The Fifth Affiliated Hospital, Guangzhou Municipal and Guangdong Provincial Key Laboratory of Molecular Target & Clinical Pharmacology, the NMPA and State Key Laboratory of Respiratory Disease, School of Pharmaceutical Sciences, Guangzhou Medical University, Guangzhou 511436, China

## Abstract

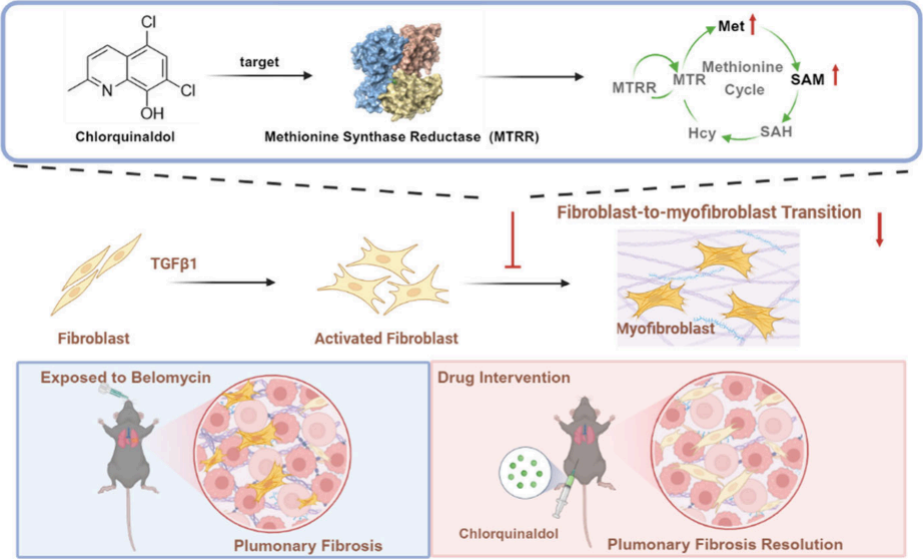

Idiopathic pulmonary fibrosis (IPF) is a progressive interstitial lung disease with limited treatment options. Thus, it is essential to investigate potential druggable targets to improve IPF treatment outcomes. By screening a curated library of 201 small molecules, we have identified chlorquinaldol, a known antimicrobial drug, as a potential antifibrotic agent. Functional analyses have demonstrated that chlorquinaldol effectively inhibits the transition of fibroblasts to myofibroblasts *in vitro* and mitigates bleomycin-induced pulmonary fibrosis in mice. Using a mass spectrometry-based drug affinity responsive target stability strategy, we revealed that chlorquinaldol inhibited fibroblast activation by directly targeting methionine synthase reductase (MTRR). Decreased MTRR expression was associated with IPF patients, and its reduced expression *in vitro* promoted extracellular matrix deposition. Mechanistically, chlorquinaldol bound to the valine residue (Val-467) in MTRR, activating the MTRR-mediated methionine cycle. This led to increased production of methionine and *s*-adenosylmethionine, counteracting the fibrotic effect. In conclusion, our findings suggest that chlorquinaldol may serve as a novel antifibrotic medication, with MTRR-mediated methionine metabolism playing a critical role in IPF development. Therefore, targeting MTRR holds promise as a therapeutic strategy for pulmonary fibrosis.

## Abstract 2

We identified MTRR as a novel target of Chlorquinaldol, which inhibits fibroblast activation and mitigates pulmonary fibrosis by binding to the valine residue (Val-467) in MTRR.

## Introduction

Idiopathic pulmonary fibrosis (IPF) is a chronic, progressive, and ultimately fatal interstitial lung disease. The primary characteristic of IPF is the excessive deposition of the extracellular matrix (ECM), destroying lung architecture. This leads to irreversible loss of lung function, respiratory failure, and ultimately death.^1,2^ The global prevalence of IPF is estimated to be around 3 million people, with the incidence increasing significantly with age.^3^ Currently, the FDA-approved drugs for the treatment of progressive pulmonary fibrosis in both IPF and non-IPF are Ofev (Nintedanib) and Esbriet (Pirfenidone), which work by inhibiting tyrosine kinases and the TGF-β pathway, respectively.^4,5^ While these medications can reduce the rate of decline in forced vital capacity and the risk of acute exacerbation, their effects on the overall progression of the disease and mortality are not particularly impressive.^6^ Moreover, these drugs are associated with significant drug-related adverse events, such as gastrointestinal problems, leading to dose reductions or discontinuation in nearly 50% of patients within one year.^7,8^ Therefore, there is an urgent need for new therapeutic approaches with novel mechanisms for treating IPF patients. Further research is needed to explore new druggable targets that can improve treatment outcomes for IPF, and compounds from the FDA-approved drug library can serve as valuable tools in identifying these new targets.

While the exact cause of IPF remains unclear, it is known that fibroblasts play a crucial role in the development of pulmonary fibrosis, making them promising targets for therapeutic interventions. When the lungs are damaged, fibroblasts from various sources become activated, transforming into myofibroblasts.^9^ These myofibroblasts are responsible for the excessive production, deposition, and remodeling of ECM proteins, which can result in dysfunctional healing.^10^ Inhibiting the transition of lung fibroblasts to myofibroblasts may offer potential benefits in treating IPF. This transition involves complex processes such as metabolic changes, epigenetic modifications, and transcriptional regulation.^11,12^ Therefore, targeting these metabolic and epigenetic pathways with specific compounds may help to inhibit lung fibroblast-to-myofibroblast transition (FMT) and effectively treat IPF.

In the present study, by performing phenotypic screening, we found that chlorquinaldol (CQD) inhibited FMT characteristics by reducing cell proliferation and ECM protein deposition. With a drug affinity responsive target stability (DARTS) assay, methionine synthase reductase (MTRR) was identified as the direct target of CQD in fibroblast. Mechanistically, CQD promotes the production of methionine and *s*-adenosylmethionine (SAM), which then inhibits fibroblast activation. Therefore, we discovered that CQD could potentially serve as a novel antifibrotic medication and identified MTRR as a target for IPF treatment by employing CQD as a molecular probe.

## Results and Discussion

### Chlorquinaldol Suppress the Proliferation and Differentiation of Fibroblasts *In Vitro*

**Figure 1:**
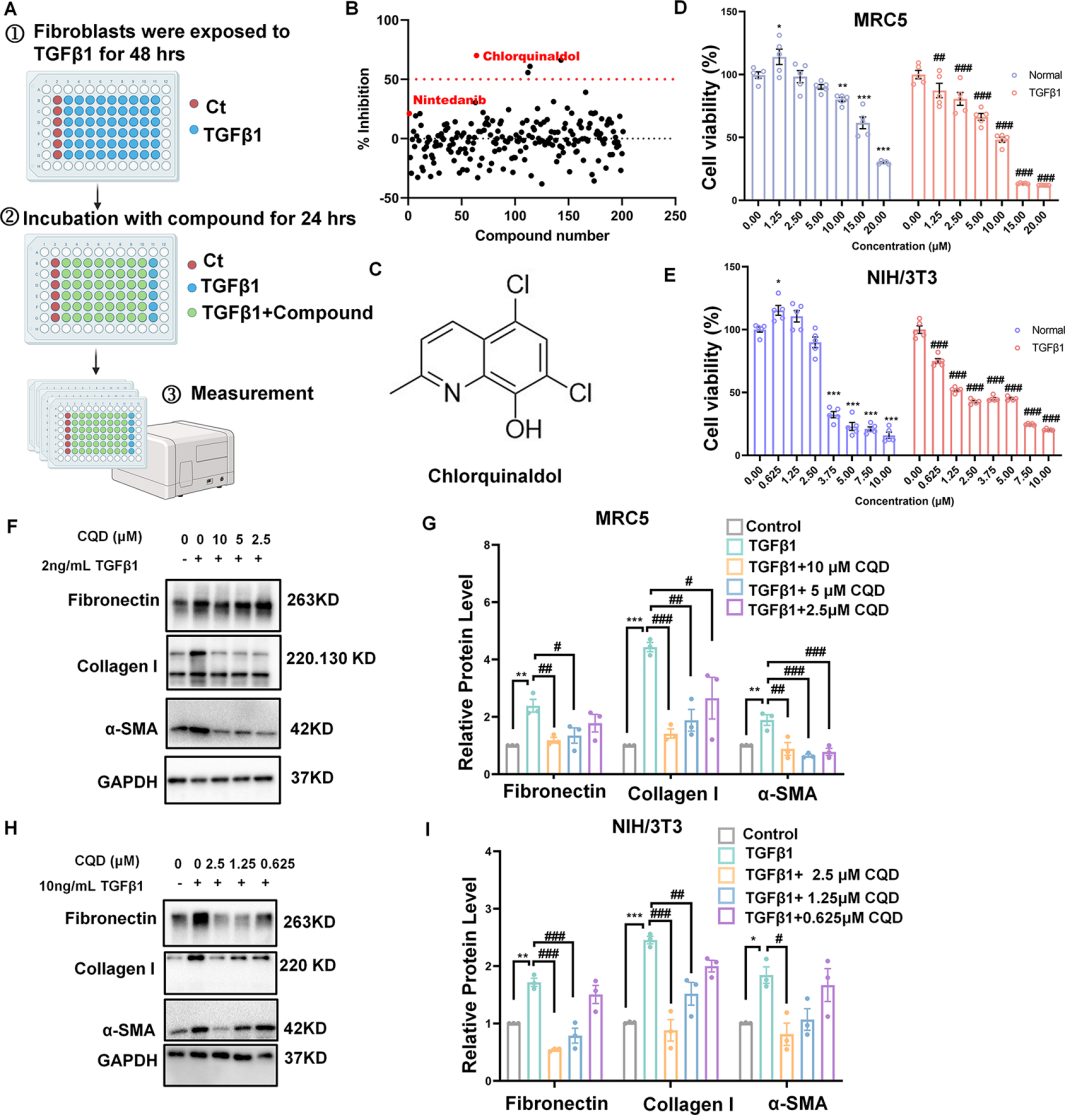
Inhibitory effects of chlorquinaldol on the proliferation and differentiation of pulmonary fibroblasts. (A) Schematic of a phenotypic screening procedure for antifibrotic agent discovery. Normal murine fibroblasts (NIH/3T3) were seeded in 96-well plates and pretreated with 10 ng/mL transforming growth factor-β1 (TGFβ1) for 48 h, followed by 24 h of exposure to 10 μM test compounds or 0.1% DMSO. Proliferation was measured via CCK8 assay to assess the antifibrotic potential of the compounds. (B) The CCK-8 assay illustrates the cell viability in response to compound exposure. Notably, four compounds significantly reduced TGFβ1-stimulated fibroblast proliferation by at least 50%. (C) The chemical structure of chlorquinaldol (CQD), characterized as 5,7-dichloro-2-methyl-8-hydroxyquinoline, a known antibacterial agent, is depicted. (D, E) Both quiescent and TGFβ1-activated MRC5 and NIH/3T3 cells were incubated with a range of CQD concentrations or DMSO vehicles for 72 h. Cell proliferation was then evaluated using the CCK-8 assay. (F, G) Following a 24 h treatment with DMSO or CQD, MRC5 cells were subjected to Western blot analysis to determine the protein expression levels of fibronectin, collagen I, and α-SMA, with GAPDH serving as the loading standard. Experiments were performed in triplicate (*n* = 3). (H, I) Western blot analysis was similarly conducted to assess the protein expression of fibronectin, collagen I, and α-SMA in NIH/3T3 cells, using GAPDH as the internal control. Each condition was replicated three times (*n* = 3). Data are represented as the mean ± standard error of the mean (SEM). Statistical significance is indicated for comparisons against unstimulated control with * for *p* < 0.05, ** for *p* < 0.01, and *** for *p* < 0.001 and against TGFβ1 alone with ^#^ for *p* < 0.05, ^##^ for *p* < 0.01, and ^###^ for *p* < 0.001.

The TGFβ pathway plays a key role activating fibroblasts, leading to fibrosis.^13^ In order to identify inhibitors of pulmonary fibrosis, we induced proliferation of mouse embryonic fibroblasts (NIH/3T3) with TGFβ1 to establish the cell model, and then conducted screening experiments. We screened 201 compounds from our in-house chemical library to assess their ability to inhibit fibroblast proliferation within 24 h (Figure 1A), with specific information available in Table S1. Using a threshold of more than 50% reduction in proliferation, we identified four noteworthy compounds. Remarkably, these candidate compounds are FDA-approved drugs, including CQD (Figure 1B, C), pemetrexed disodium hernipenta hydrate, ribociclib (LEE011 succinate), and lasofoxifene tartrate. A comprehensive literature review indicated that CQD, in particular, has not been previously investigated for its antifibrotic properties, thereby directing our attention to its potential.

To assess whether the inhibitory effect of CQD on fibroblasts proliferation is due to its toxicity, we initially evaluated cell viability in human fetal lung fibroblasts (MRC5) and NIH/3T3 following 72 h of exposure to various concentrations of CQD, with or without TGFβ1 stimulation. The results showed that the IC_50_ values for CQD were 16.22 μM for nonactivated MRC5 cells and 7.15 μM for activated MRC5 cells. The IC_50_ for NIH/3T3 cells was 3.61 μM (nonactivated) and 2.11 μM (activated) (Figure 1D, E). These results indicated that the inhibitory effect of CQD on proliferation is not due to cytotoxicity. Furthermore, Western blot analysis demonstrated that CQD reduced the expression of myofibroblasts biomarker α-smooth muscle actin (α-SMA) and ECM protein molecules, namely Fibronectin, Collagen I, in a dose-dependent manner in both MRC5 and NIH/3T3 cell lines (Figure 1F–I). These results suggest that CQD may exert its antifibrotic effects by inhibiting the FMT process, which is a central mechanism in fibrotic tissue remodeling.

### Chlorquinaldol Prevents Lung Fibrosis in BLM-Induced Lung Remodeling

**Figure 2:**
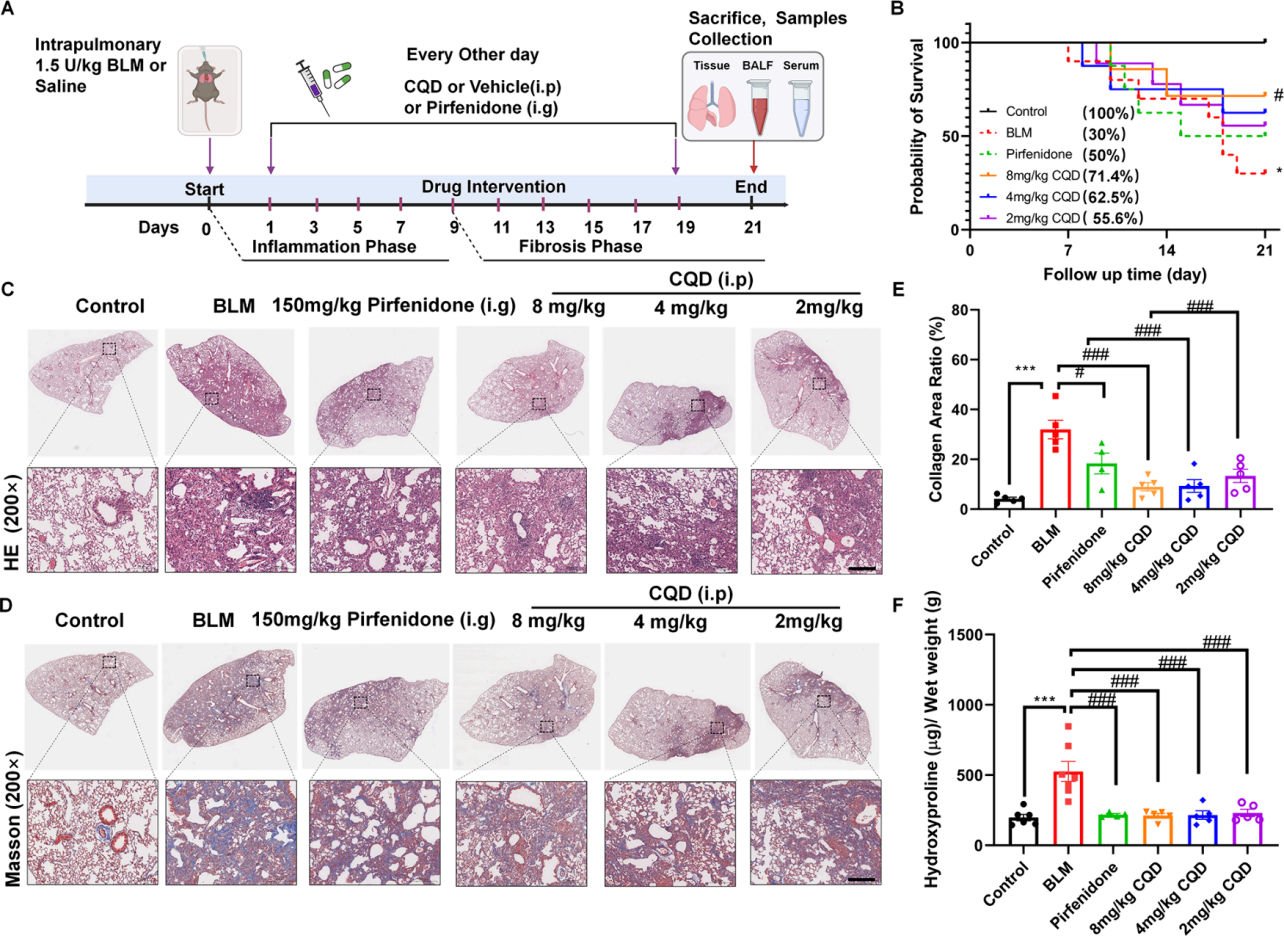
Protective effect of chlorquinaldol on bleomycin-induced pulmonary fibrosis. (A) Schematic of the preventive treatment protocol for lung fibrosis in animal models. (B) Survival curve for mice over 21 days following BLM challenge (*n* = 6–10). (C) HE stained lung tissue sections from mice on day 21 after BLM exposure. The inset displays a zoomed area (200× magnification), with a 200 μm scale bar (*n* = 4–6). (D) Representative images of Masson’s trichrome staining in lung tissue; the inset zooms in on a detailed area (200 × ), with a 200 μm scale bar (*n* = 4–6). (E) Assessment of the collagenous area ratio from Masson’s trichrome-stained sections. (F) Quantification of hydroxyproline levels in the right lung lobes. Data presented as mean ± SEM for 4–7 animals per group. Significance is indicated by *p < 0.05, **p < 0.01, and ***p < 0.001 for comparisons with the control and ^#^*p* < 0.05, ^##^*p* < 0.01, and ^###^*p* < 0.001 for other group comparisons as indicated.

To evaluate the potential protective effects of CQD *in vivo*, a single intratracheal instillation of bleomycin (BLM) was given at a dosage of 1.5 U/kg. CQD, dissolved in a DMSO/PBS solution, or the solvent alone (as control) was administered intraperitoneally to mice every 48 h, starting from Day 1 after BLM administration and continuing until the end of the study. Pirfenidone, an established antifibrotic drug, was administered orally every 48 h as a positive control for preventive treatment, as depicted in Figure 2A. Our findings indicated that CQD significantly improved the survival rate of mice with BLM-induced pulmonary fibrosis in a dose-dependent manner (Figure 2B).

Histological analysis using Hematoxylin and Eosin (HE) and Masson’s trichrome staining showed that the control group maintained normal pulmonary architecture with distinct alveolar structures and no septal thickening. Conversely, the BLM group displayed significant architectural derangement, pronounced septal thickening, evident pulmonary consolidation, and extensive deposition of collagen fibers (stained blue). Consistently, treatment with CQD markedly improved alveolar space collapse and septal thickening, reduced infiltration of inflammatory cells, alleviated lung tissue damage, and reduced deposition of collagen fibers (Figure 2C, D). Notably, the high dose of CQD (8 mg/kg) demonstrated superior therapeutic efficacy compared to pirfenidone, as illustrated in Figure 2E. Additionally, a decrease in hydroxyproline content was observed within the lung tissue after CQD treatment (Figure 2F). Immunofluorescence assays also demonstrated a significant reduction in the expression of fibrosis-associated proteins, such as Type I Collagen (COL1A1) and α-smooth muscle actin (α-SMA), following CQD treatment (Figure S1).

In recognition of the pivotal role of inflammation in the progression of lung fibrosis,^14^ we measured levels of inflammatory cytokines, such as IL-6, oncostatin M (OSM), IL-1β, and TGFβ1, in bronchoalveolar lavage fluid and serum samples from the mice using enzyme-linked immunosorbent assay (ELISA). A dose-dependent decrease in IL-6 and OSM levels was observed in the CQD-treated group, while no significant alterations were detected in IL-1β and TGFβ1 levels (Figure S2). Subsequent pharmacokinetic study was conducted following an intraperitoneal dose of 8 mg/kg in C57BL/6J mice. According to Table S1, CQD exhibited a favorable pharmacokinetic profile, characterized by a reasonable half-life (*T*_1/2_ = 1.2 h) and an acceptable maximum plasma concentration (*C*_max_) of 10.83 ng/mL (47.49 nM). We observed that the CQD plasma concentration is lower than that described in the cell culture study. Given the varying drug sensitivities of different types of fibroblasts and the diverse cell culture conditions, we believe that the effective concentrations of CQD observed in animal studies and cell culture experiments are not directly comparable. Furthermore, organ toxicity assays revealed no significant pathological changes in the heart, kidney, liver, or spleen following CQD administration, confirming its favorable safety profile within the therapeutic dosage range of 2 to 8 mg/kg (Figure S2).

### Chlorquinaldol Reverses Lung Fibrosis in BLM-Induced Lung Remodeling

**Figure 3:**
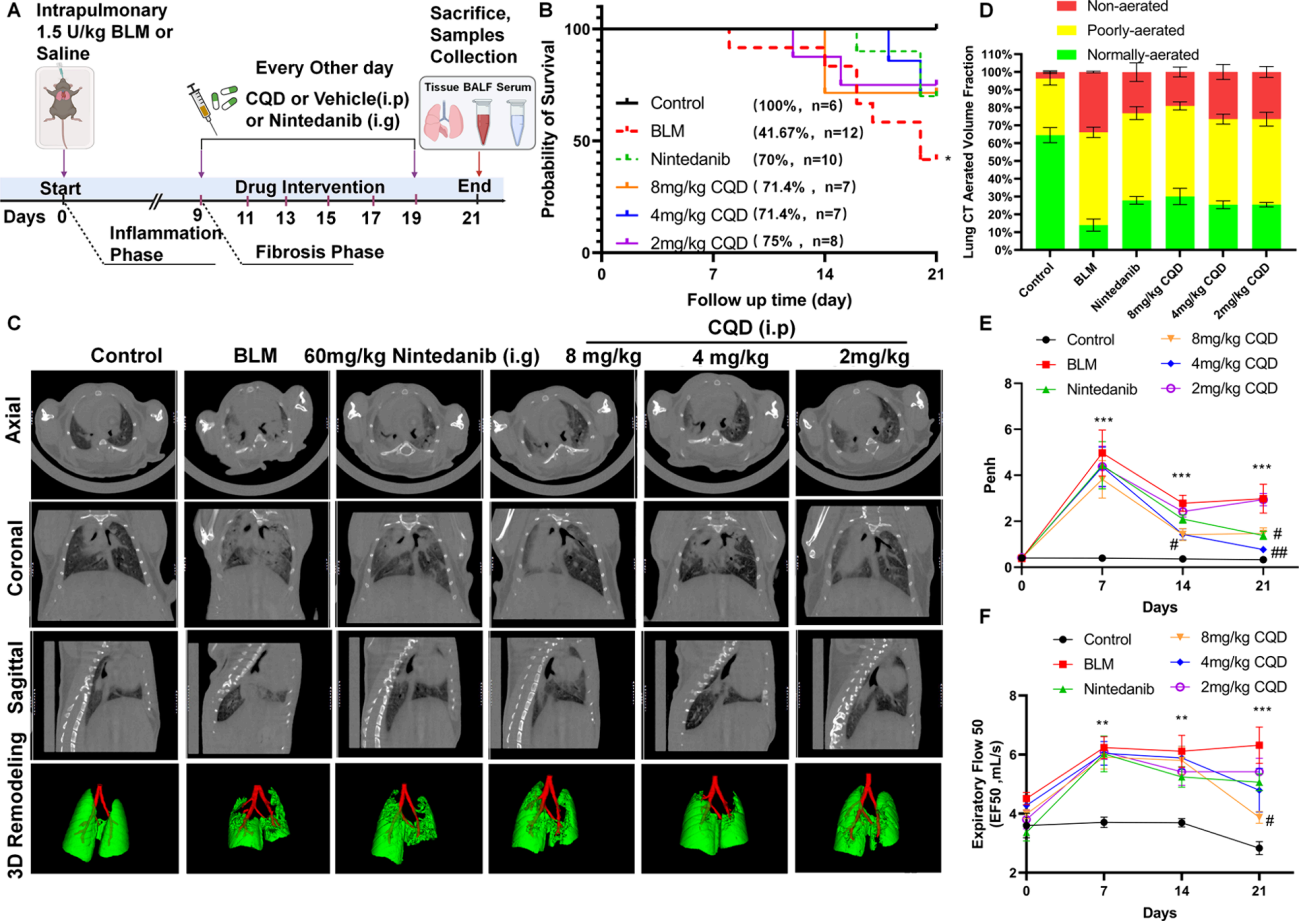
Chlorquinaldol ameliorates pulmonary fibrosis and lung ventilation. (A) Treatment protocol for the pulmonary fibrosis mouse model, including the administration timeline for CQD or nintedanib. The first 9 days of post-BLM induction represent the inflammatory phase, followed by the fibrotic phase. (B) The survival rates of mice within the 21-day BLM model (*n* = 6–12). (C) Representative micro-CT images of the whole lung on the 21st day, featuring axial, coronal, and sagittal views, as well as three-dimensional reconstructions. All images of the right mainstem bronchus bifurcation were selected to ensure consistent anatomical comparison. (D) Analysis of the lung volume ventilation fraction in mice, with green indicating normally aerated areas (−860 to −435 HU), yellow representing poorly aerated areas (−434 to −121 HU), and red signifying nonaerated regions (−120 to +121 HU). Data are shown as mean ± SEM, with *n* = 3–5 per group. (E, F) The dynamic changes in the airway constriction index Penh and the midexpiratory flow rate EF_50_, each presented as mean ± SEM (*n* = 3–12). Statistical significance is denoted by *p < 0.05, **p < 0.01, and ***p < 0.001 for comparisons with the control group and ^#^*p* < 0.05, ^##^*p* < 0.01, and ^###^*p* < 0.001 for comparisons with the BLM group.

CQD has exhibited a wide range of pharmacological effects, extending beyond its original classification as an antimicrobial to encompass antifungal, antitubercular, antiparasitic, antiviral, anti-inflammatory, and antitumor properties.^15−19^ To verify the direct effects of CQD on the fibrotic phase, independent of its anti-inflammatory activity, mice exposed to BLM received intraperitoneal CQD treatment every 48 h from day 9 post-BLM exposure, or oral administration of nintedanib. Lung function was monitored on days 0, 7, 14, and 21, complemented by micro-CT imaging for comprehensive lung assessment and tissue collection for subsequent analyses on day 21 (Figure 3A).

**Figure 4:**
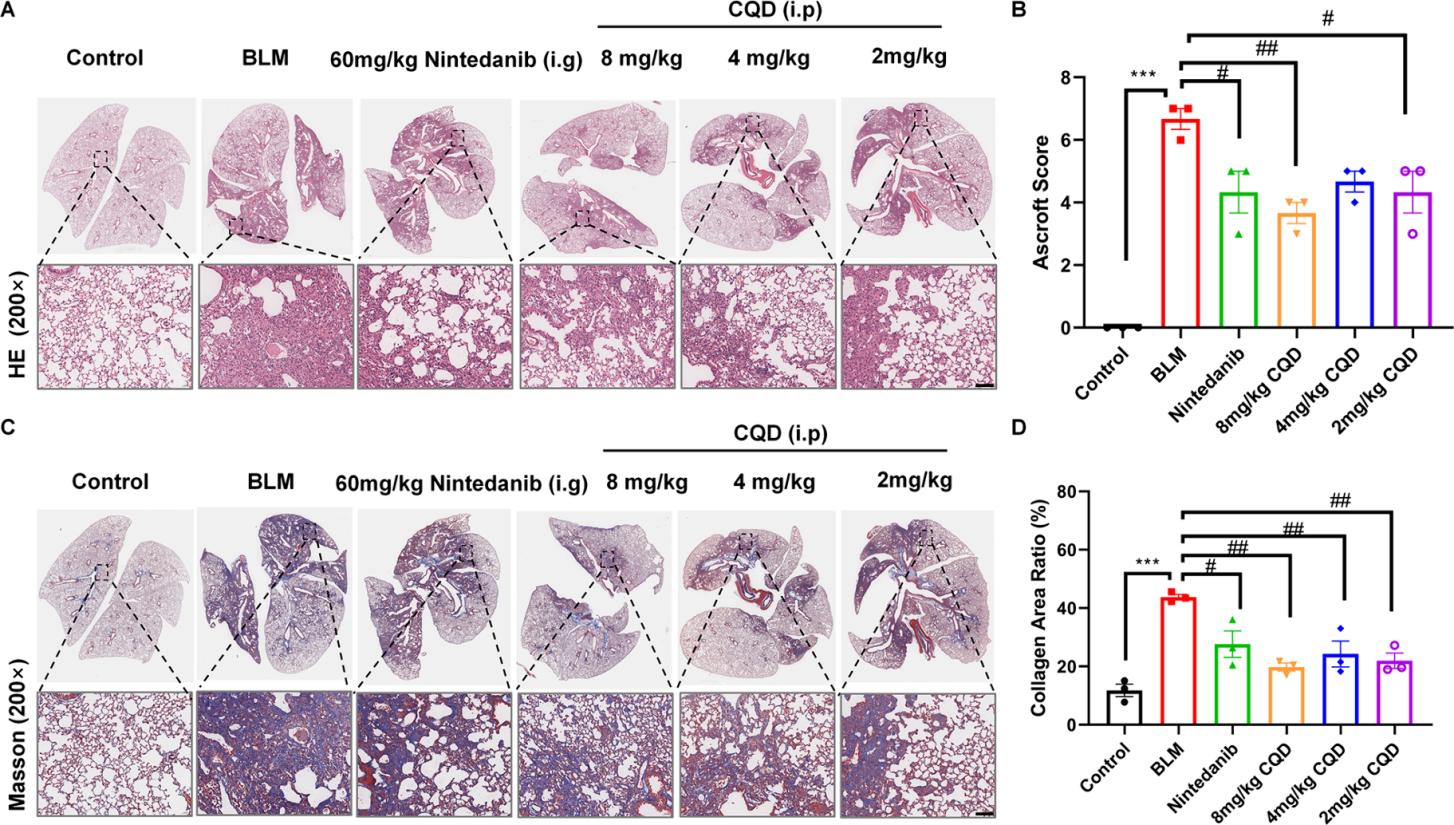
Chlorquinaldol improved alveolar architecture and reduced interstitial collagen deposition. (A) Representative images of whole lung HE staining on the 21st day. (B) Pulmonary fibrosis scores based on the Ashcroft scoring system. (C) Representative Masson’s trichrome staining of entire lung tissue from the mice. (D) Quantitative analysis of collagen content as determined by Masson’s staining. Scale bar: 100 μM. Data is expressed as mean ± SEM (*n* = 3). Significance is denoted by *p < 0.05, **p < 0.01, ***p < 0.001 compared to the control and ^#^*p* < 0.05, ^##^*p* < 0.01, ^###^*p* < 0.001 for comparisons with the BLM group.

The results showed that CQD therapy significantly improved survival in mice, with efficacy comparable to that of nintedanib (Figure 3B). Micro-CT imaging, a vital diagnostic and therapeutic efficacy assessment tool for IPF,^20−24^ revealed a decreased distribution of fibrotic lesions in lung tissue following CQD treatment (Figure 3C). Quantitative analysis of micro-CT scans showed a significant increase in normally aerated lung volume in the 8 mg/kg CQD group, similar to the effects observed in the nintedanib group (Figure 3D). Pulmonary function assessments also indicated this dosage of CQD markedly alleviated airway obstruction, as evidenced by the lowered Penh and EF_50_ values, similar to the functional improvement seen with nintedanib treatment (Figure 3E-F). Histopathological analysis showed improvements in alveolar architecture and reduced interstitial collagen deposition after CQD treatment (Figure 4A-D). Collectively, these findings indicate that CQD might alleviate pulmonary fibrosis severity via its inherent antifibrotic mechanisms.

### Methionine Synthase Reductase Is a Novel Target of Chlorquinaldol

**Figure 5:**
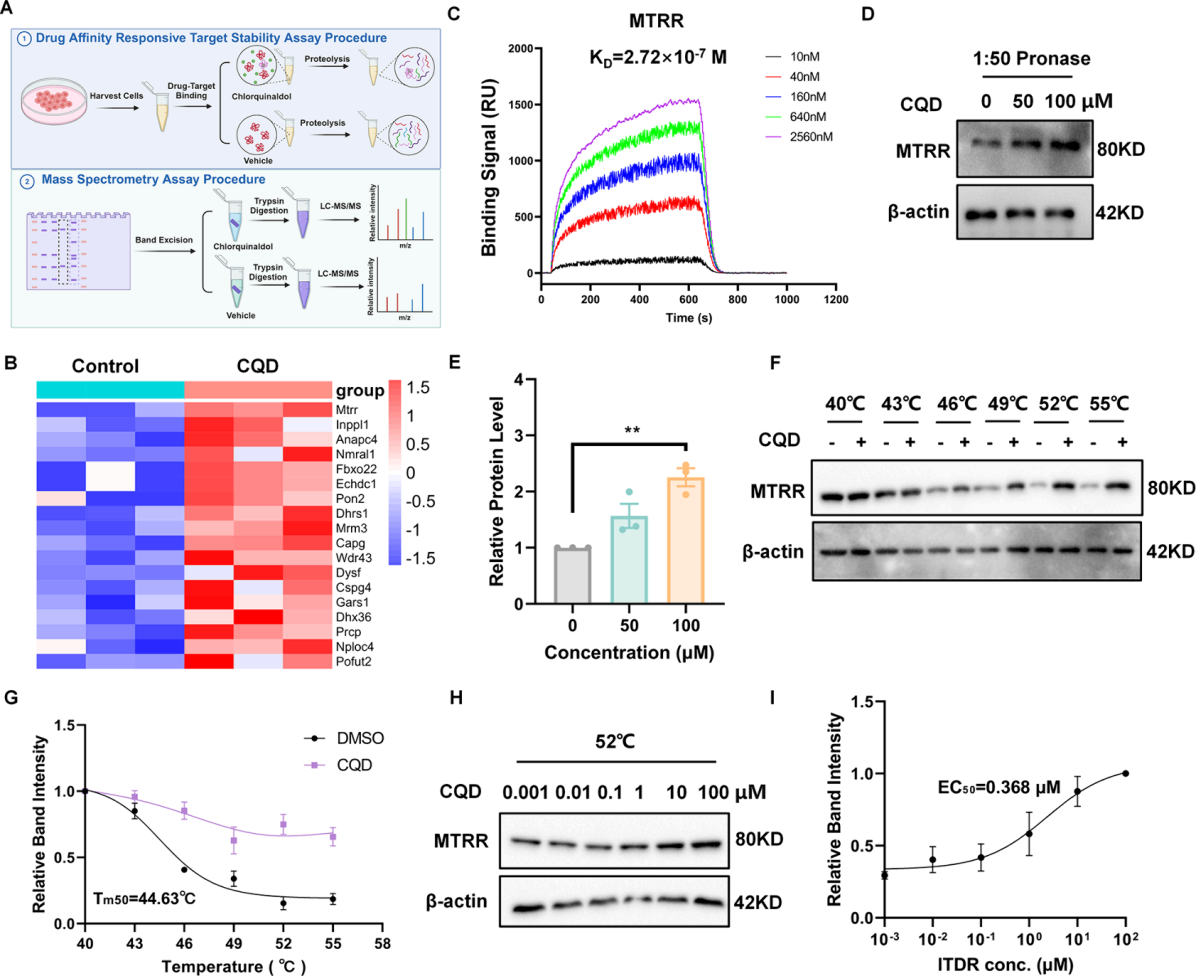
Chlorquinaldol directly targets MTRR protein. (A) Schematic of the DARTS/MS strategy for discovering potential chlorquinaldol binding proteins. (B) Heatmap of 18 candidate targets with differential expression levels, as determined by mass spectrometry. (C) SPR measures the binding affinity of chlorquinaldol to MTRR protein. (D, E) Immunoblots of MTRR levels in TGFβ1-activated NIH/3T3 cells with chlorquinaldol treatment and subsequent Pronase digestion. (F, G) CETSA melt response and related curves to assess the thermostability between chlorquinaldol and MTRR. (H, I) Isothermal dose response (ITDR) and its curve indicating the binding thermodynamics of chlorquinaldol. Data are mean ± SEM (*n* = 3), with statistical significance marked by *p < 0.05, **p < 0.01, ***p < 0.001 versus control. Abbreviations: MTRR, methionine synthase reductase; DARTS, drug affinity responsive target stability assay; SPR, surface plasmon resonance; CETSA, cellular thermal shift assay; CQD, chlorquinaldol.

To investigate the direct target and molecular mechanism responsible for the antipulmonary fibrosis effects of CQD, we conducted drug affinity responsive target stability (DARTS) and mass spectrometry assay (Figure 5A), which identified 78 differentially expressed proteins as potential targets of CQD (Figure 5B; Table S2). From this pool, MTRR, inositol polyphosphate-specific phosphatase 1 (INPPL1), and glycyl-tRNA Synthetase 1 (GARS1) were selected for in-depth analysis. Surface plasmon resonance (SPR) analysis revealed that CQD binds to MTRR with high affinity, as indicated by an association rate constant (Ka) of 2.06 × 10^4^ M/s and a dissociation rate constant (*K*_d_) of 5.6 × 10^–3^ 1/s, resulting in an equilibrium dissociation constant (K_D_) of 2.72 × 10^–7^ M (Figure 5C). In comparison, the K_D_ values for the CQD interactions with INPPL1 and GARS1 were 9.44 × 10^–3^ M and 6.16 × 10^–4^ M, respectively, denoting a weaker interaction with CQD (Figure S3).

To validate the direct binding of MTRR to CQD, we performed protein immunoblotting of DARTS samples using various concentrations of CQD in cell lysates. The results showed that CQD effectively shielded MTRR from proteolytic degradation by Pronase, confirming MTRR as a direct target of CQD (Figure 5D-E). To further confirm the association between MTRR and CQD, we utilized the cellular thermal shift assay (CETSA) to assess the thermal stability of MTRR across various temperatures and CQD concentrations. The assay revealed a marked shift in the thermal melting curve for CQD-treated samples, as compared to DMSO control (Figure 5F-G), and indicated that CQD stabilizes MTRR in a concentration-dependent manner, with an effective concentration (EC_50_) of 0.368 μΜ (Figure 5H–I). These collective results suggest a direct interaction between CQD and MTRR, which may contribute to the antifibrotic activity of CQD.

### Chlorquinaldol Interacts with the FAD-Binding Domain of Methionine Synthase Reductase

**Figure 6:**
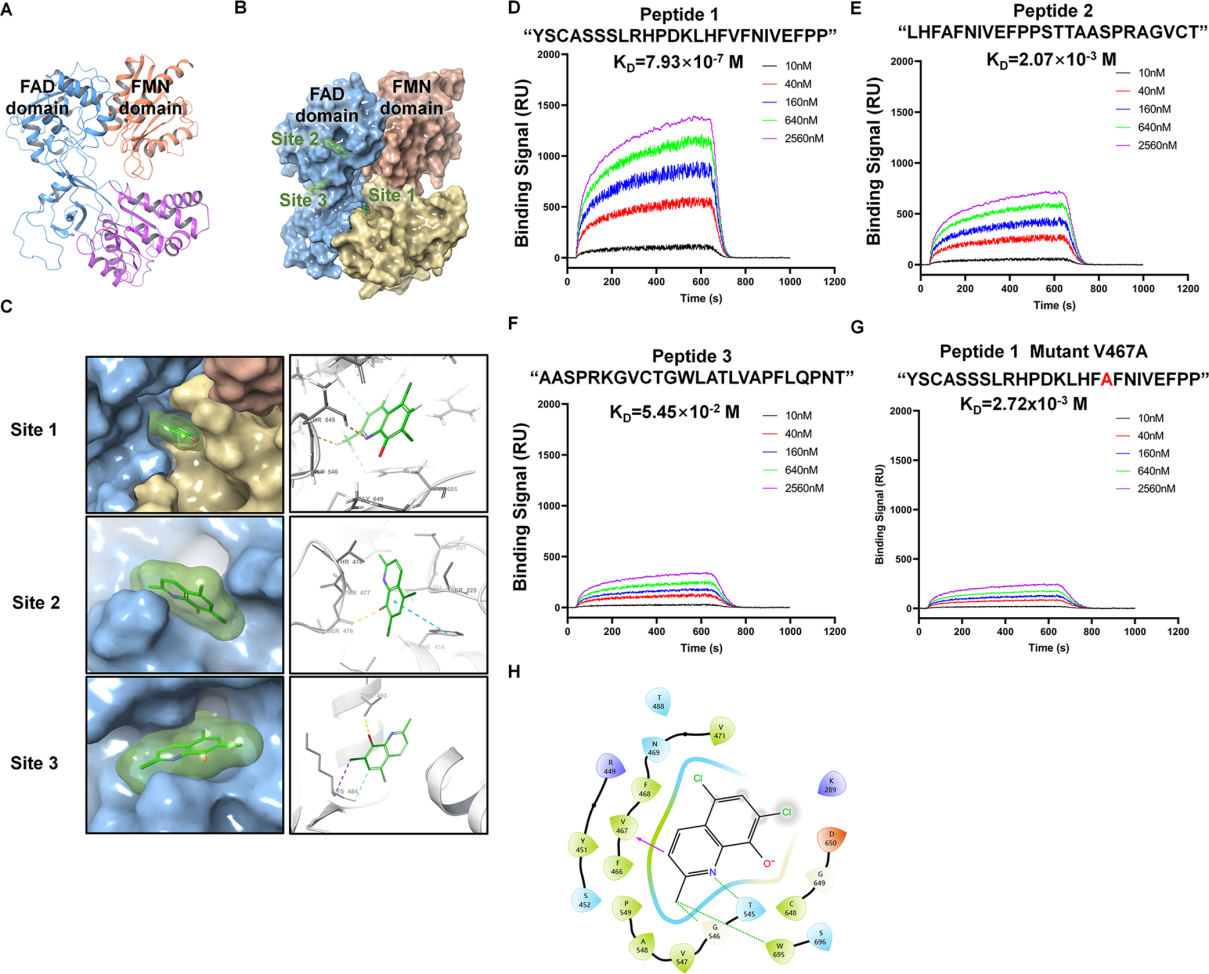
Chlorquinaldol exerts antifibrotic activity by directly interacting with the FAD domain of the MTRR protein. (A) Cartoon depiction of the structure of murine methionine synthase reductase (MTRR), featuring FMN and FAD domains connected by a flexible hinge. (B) Three-dimensional representation demonstrating chlorquinaldol’s top three binding sites within the MTRR protein architecture. (C) Detailed 3D interaction map of chlorquinaldol with the MTRR protein, alongside a local secondary structure binding interaction diagram. (D–G) SPR analysis detecting the binding of four peptides (peptide 1, 2, and 3 and peptide 1 mutant V467A) to chlorquinaldol. (H) Molecular docking simulation revealing the chlorquinaldol–MTRR binding interface, with interactions indicated by purple arrows and noncovalent distance interactions indicated by green dashed lines. Abbreviation: FMN, flavin mononucleotide; FAD, flavin adenine dinucleotide.

The MTRR gene, consisting of 15 exons and 14 introns, is located in the P15.2–15.3 region of chromosome 5 and produces transcripts ranging in length from 108 bp to 5 kb. MTRR encodes an enzyme with flavin reductase activity that shares a similar N-terminal structure to flavin oxidases, consisting of an FMN domain connected to the C-terminal NADP(H)-flavin oxidoreductase-like FAD domain and NADP(H)-binding site via a hinge region.^25−28^ To identify the specific binding site of CQD on MTRR, the Maestro software was utilized for homology modeling of the murine MTRR protein structure (Figure 6A), followed by structural optimization and molecular docking. By analyzing various conformations of CQD and its binding modes with MTRR, we identified the three most stable binding sites with the lowest docking free energy for further investigation (Figure 6B). The molecular docking models predicted the sequences of the binding epitopes on MTRR as follows: epitope peptide 1: “YSCASSSLRHPDKLHFVFNIVEFPP”; epitope peptide 2: “LHFAFNIVEFPPSTTAASPRAGVCT”; and epitope peptide 3: “AASPRKGVCTGWLATLVAPFLQPNT” (Figure 6C).

To validate these interactions, we synthesized the corresponding peptides and conducted SPR assays. The data showed a high affinity of CQD for peptide 1 with a K_D_ of 7.93 × 10^–7^ M, a weaker interaction with peptide 2 (K_D_ = 2.07 × 10^–3^ M), and no interaction with peptide 3 (K_D_ = 5.45 × 10^–2^ M) (Figure 6D-F). Subsequent analysis of the molecular docking results suggested that CQD interacts with the valine residue (Val 467) within the FAD-binding domain of MTRR, with a bond length of 2.51 Å and a free energy of −0.4105 kcal/mol. Mutations at epitope peptide 1 (V467A) were found to affect CQD binding, resulting in a K_D_ of 2.72 × 10^–3^ M (Figure 6G). Additionally, CQD appears to interact with MTRR through noncovalent bonds with threonine (Thr 545), glycine (Gly 546), and tryptophan (Trp 695) residues, with corresponding bond lengths of 2.22 Å, 2.18 Å, and 1.97 Å, respectively (Figure 6H). Molecular dynamics simulations confirmed the stability of the CQD-MTRR complex in an aqueous environment, with no significant decomposition or aggregation observed for either CQD or MTRR (Figure S4). These results shed light on the specific interaction between CQD and MTRR, which could be crucial for developing targeted therapies.

### Chlorquinaldol Activates MTRR to Suppress Fibroblast Activation through Methionine Cycle Regulation

**Figure 7:**
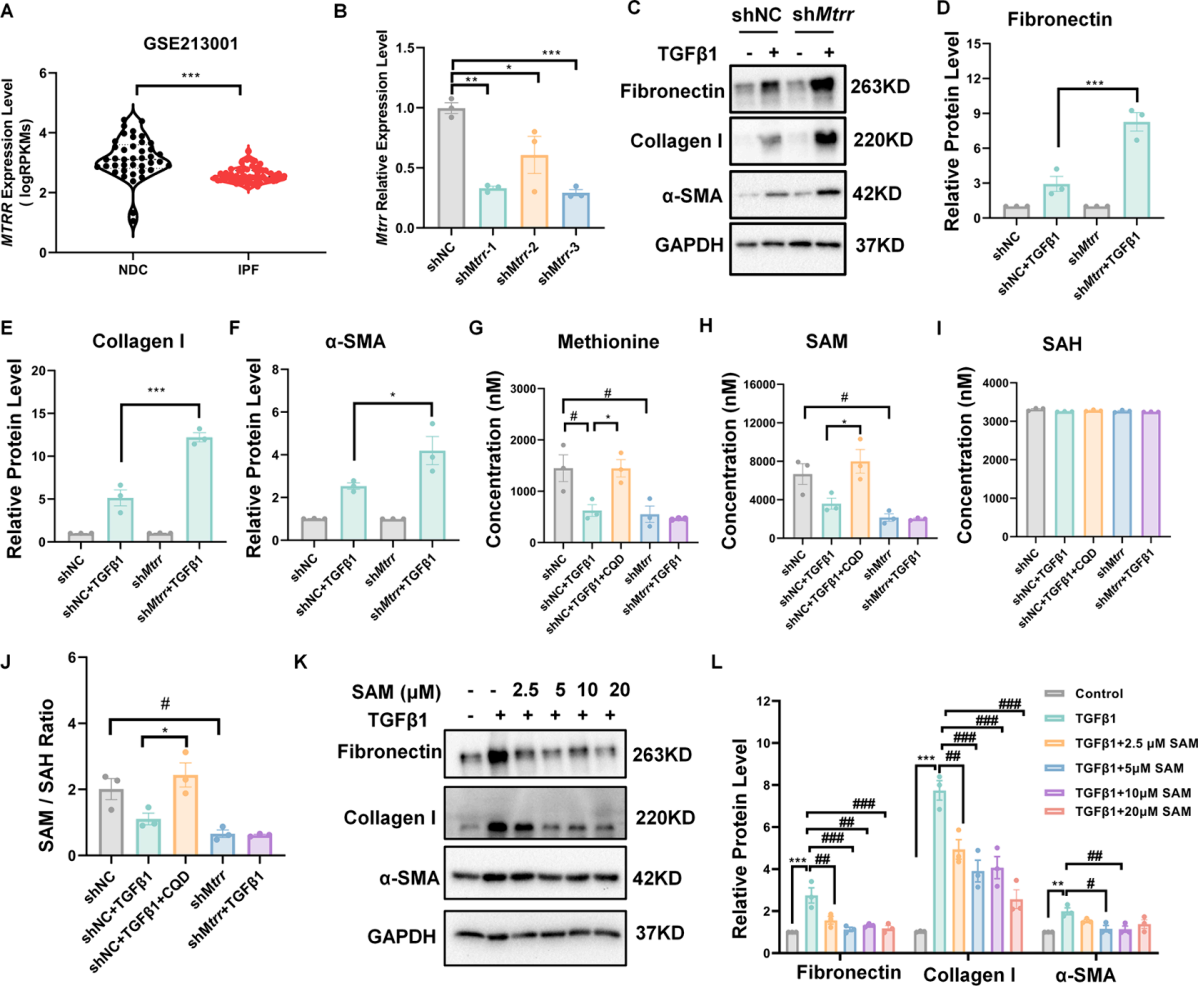
Chlorquinaldol promotes methionine and *s*-adenosyl methionine (SAM) accumulation via MTRR to inhibit fibrosis. (A) A violin plot illustrates the expression levels of methionine synthase reductase (MTRR) in lung tissues from idiopathic pulmonary fibrosis (IPF) patients, with data obtained from the GEO data set GSE213001. (B) qPCR analysis assesses the efficiency of *Mtrr* knockdown in NIH/3T3 cells. (C–F) Western blot (WB) analysis measures the protein expression levels of fibronectin, collagen I, and α-smooth muscle actin (α-SMA) in *Mtrr* knockdown cells following TGFβ1 stimulation. (G–J) HPLC/MS was utilized to quantify intracellular methionine, SAM, and SAH levels. (K–L) NIH/3T3 cells are pretreated with varying concentrations of SAM for 24 h before TGFβ1 stimulation, and WB is used to assess the protein expression levels of fibronectin, collagen I, and α-SMA. Data are presented as mean ± SEM (*n* = 3). Significance is denoted by *p < 0.05, **p < 0.01, and ***p < 0.001 compared to the control. Abbreviations: SAM, *s*-adenosylmethionine; SAH, *s-*adenosylhomocysteine.

Functionally, MTRR acts as a crucial cofactor for methionine synthase (MTR), facilitating the conversion of methyltetrahydrofolate and homocysteine to tetrahydrofolate and methionine catalyzed by MTR.^29,30^ Aberrant MTRR expression has been linked to cardiovascular diseases and multidrug resistance in ovarian cancer.^31,32^ However, the role of MTRR in pulmonary fibrosis and its underlying molecular mechanisms remains uncertain. Due to the unclear clinical relevance of MTRR in IPF, we analyzed MTRR expression in lung tissues from a cohort of 41 normal individuals and 62 IPF patients retrieved from the GEO database (GSE213001). We observed a significant downregulation of MTRR in IPF tissues, suggesting an inverse correlation with disease progression (Figure 7A). To further elucidate the biological role of MTRR in fibrosis, we employed shRNA interference to suppress MTRR expression in NIH/3T3 cells (Figure 7B). Western blot analysis revealed that MTRR knockdown cells showed elevated levels of ECM components, such as Fibronectin and Collagen I, as well as myofibroblast markers like α-SMA, particularly under TGFβ1 stimulation (Figure 7C–F). These findings suggest that CQD could potentially attenuate fibrosis progression by activating MTRR, thus suppressing fibroblast phenotypic changes.

We further explored the regulatory effect of CQD on MTRR by measuring MTRR mRNA and protein levels in NIH/3T3 cells after CQD treatment. Quantitative PCR and Western blot analyses showed no significant changes in MTRR levels, indicating that the antifibrotic effect of CQD is not mediated by altering MTRR expression (Figure S5). Subsequently, a significant elevation in MTRR activity was observed post-treatment with CQD, as illustrated by the Cytochrome c Reduction Assay (Figure S6). This suggests that CQD may inhibit fibrosis by modulating MTRR enzymatic activity. A limitation of our study is the absence of *Mtrr* knockout models during the identification of MTRR as a potential target for CQD in treating lung fibrosis. Future investigations will focus on elucidating the relationship between MTRR and lung fibrosis utilizing *Mtrr* knockout cell and animal models.

MTRR plays a pivotal role in folate and methionine metabolism, but the specific metabolic pathway through which it modulates fibrosis remains unclear. We initially assessed whether folate contributes to fibrosis using Western blot analysis. The results showed that folate does not significantly affect fibroblast differentiation (Figure S7), implying that MTRR might mediate fibrosis through methionine metabolism rather than folate metabolism. Employing high-performance liquid chromatography and mass spectrometry (HPLC/MS), we quantified methionine cycle metabolites and intriguingly found that CQD increased intracellular methionine and SAM levels, while maintaining *s-*adenosylhomocysteine (SAH) levels, thereby augmenting the SAM/SAH ratio and indicating enhanced methylation capacity. Conversely, MTRR suppression exhibited decreased levels of methionine and SAM, with an unchanging SAH level, leading to a decreased SAM/SAH ratio and methylation potential (Figure 7G-J). Furthermore, SAM and methionine were found to reduce TGFβ1-induced expression of Fibronectin, Collagen I, and α-SMA in NIH/3T3 cells, underscoring its antifibrotic activity (Figure 7K-L and Figure S8). Furthermore, Cycloleucine, an inhibitor of SAM, reversed CQD’s inhibitory effect on TGFβ1-induced expression of Fibronectin, Collagen I, and α-SMA, suggesting that the boosted SAM production by CQD contributes to its antifibrotic activity (Figure S8). Consistent with our findings, Yoon et al. (2016) reported that SAM can alleviate airway inflammation and fibrosis in a mouse model of chronic asthma, reinforcing the antifibrotic role of SAM.^33^

The study provides preliminary evidence of the antifibrotic properties of CQD. In a murine model of pulmonary fibrosis, this FDA-approved antimicrobial agent significantly reduces mortality, improves pulmonary obstruction, and preserves lung function. It exhibits superior efficacy compared to pirfenidone and yields outcomes similar to nintedanib. The therapeutic potential of CQD may stem from its ability to suppress proliferation, fibrotic phenotypic transition, and extracellular matrix remodeling of lung fibroblasts. Mechanistic insights suggest that CQD directly targets MTRR, modulating its enzymatic function through interaction with a key valine residue in the FAD domain. This interaction increases methionine cycle activity and raises SAM levels, thus enhancing the antifibrotic effects. This discovery reveals a novel molecular mechanism underlying the potential role of CQD in IPF treatment.

Recent research has emphasized the crucial role of fibroblast amino acid metabolism, particularly methionine, proline, and arginine, in collagen synthesis, myofibroblast activation, and extracellular matrix degradation, offering potential new targets for therapeutics in fibrotic diseases.^34−38^ Methionine, an essential amino acid, plays a crucial role in various physiological processes, such as protein synthesis, serving as a precursor for the antioxidant glutathione, influencing polyamine synthesis necessary for cell division, and acting as a methyl donor in methylation processes.^39−41^ Disruptions in methionine metabolism are implicated in the worsening of pathological conditions. Methionine-restricted diets were employed in hepatic models to mimic chronic liver fibrosis, and adjustment of methionine metabolism has been demonstrated to alleviate hepatic damage.^42−44^ SAM, the active form of methionine, has been shown to reduce liver injury and fibrosis.^45^ Our study also observed that SAM can inhibit the differentiation of pulmonary fibroblasts. Numerous publications have reported the significant role of SAM in fibroblast phenotypic changes and fibrosis. For instance, Casini et al. (1989) reported that the addition of SAM to fibroblasts led to a significant decrease in collagen synthesis.^46^ In animal models, animals treated with SAM demonstrated a significant reduction in liver fibrosis in an ethanol-LPS rat model of fibrotic liver and airway fibrosis in a mouse model of chronic asthma.^33,47^ Nevertheless, Jubinville reported that deficiencies in dietary choline and/or methionine can significantly alleviate inflammatory responses and reduce the expression of key fibrotic genes such as Col1a1, Col3a1, and Elastin, thereby mitigating lung damage caused by cigarette smoke.^48^

SAM is the primary methyl donor in mammalian cells, facilitating the transfer of methyl groups to acceptor molecules like DNA, proteins, and lipids, thereby altering their structure and function.^45^ Numerous studies have demonstrated that SAM treatment leads to DNA hypermethylation, a process linked to gene expression silencing.^49^ Additionally, SAM provides protection against oxidative stress by serving as a precursor for cysteine, an essential component of glutathione, which is a primary physiological defense mechanism against reactive oxygen species.^50^ Nevertheless, the specific pathways through which CQD/methionine/SAM exerts its antifibrotic effects remain unclear in this study, which should be considered a limitation of this study. Therefore, further research is needed to comprehensively understand the role of methionine and the mechanism of SAM in pulmonary fibrosis, particularly its potential role in epigenetic methylation.

## Conclusion

We identified CQD as a novel lead compound for therapeutic applications in pulmonary fibrosis. CQD alleviates pulmonary fibrosis in mice, potentially through hydrogen bonding with the valine residue at position 467 within the FAD domain of MTRR, thus altering the spatial conformation of MTRR and subsequently activating the methionine cycle to increase the SAM levels against fibrosis. This study collectively emphasizes the repurposing of CQD as a new antifibrotic drug and identifies MTRR as a potential target for treating IPF.

## Materials and Methods

### Cell Culture

The NIH/3T3 and MRC5 cell lines were obtained from Procell (Wuhan, China) and cultured according to the manufacturer’s instructions. NIH/3T3 cells were maintained in Dulbecco’s Modified Eagle’s Medium (DMEM, Gibco, 11965092) supplemented with 10% fetal bovine serum (FBS) in a 5% CO_2_ atmosphere at 37 °C. MRC5 cells were cultured in Minimum Essential Medium (MEM, Gibco, 11090081) with 10% FBS. The NIH/3T3 cells were stimulated with a final concentration of 10 ng/mL recombinant human transforming growth factor β1 (TGFβ1, Peprotech, 100–21), while MRC5 cells were treated with 2 ng/mL.

### Cell Viability Assay

Cells were seeded at a density of 2,000 cells per well in 96-well plates using their respective media as described earlier. They were then treated with varying doses of chlorquinaldol (CQD; MedChem Express, HY-B1360) for 72 h. Absorbance was measured at 450 nm following incubation with 10 μL of Cell Counting Kit-8 (CCK-8, DOJINDO, CK04) for 2 h.

### Western Blot Analysis

Total protein was extracted from cells using RIPA buffer (Beyotime, P0013B, China) supplemented with EDTA-free protease inhibitor (ThermoFisher Scientific, A32965). According to the manufacturer’s instructions, the isolated protein was quantified with the Pierce BCA protein assay kit (ThermoFisher Scientific, 23225). Equal-quality prepared protein samples were then separated using 10% sodium dodecyl sulfate-polyacrylamide gel electrophoresis (SDS-PAGE) and transferred to a poly vinylidene fluoride (PVDF) membrane (0.2 μm, Millipore, ISEQ00010). Following blocking with 5% milk in TBS and 0.05% tween 20, the blots were probed with particular primary antibodies against α-SMA (Abcam, ab124964, 1:20000), Fibronectin (Abcam, ab45688, 1:10000), Collagen I (Abcam, ab270993, 1:1000; for mouse), Collagen I (Abcam, ab260043, 1:1000; for human), MTRR (Proteintech, 26944–1AP, 1:500), GAPDH (Servicebio, GB12002–10, 1:2000), β-actin (Sigma, A1978, 1:2000) overnight at 4 °C. The membranes were rinsed and incubated on the second day with appropriate horseradish peroxidase-conjugated (HRP) mouse or rabbit secondary antibodies (Promega, W4022 and W4011). Finally, enhanced chemiluminescent (ECL) HRP substrate (TANON, 180–506) was used to visualize the protein bands on a ChemiDoc XRS system (Bio-Rad). Quantification of protein bands was performed using Image Lab analysis software (Bio-Rad).

### Quantitative Real-Time PCR (qRT-PCR)

Total RNA was isolated using TRizol reagent (Invitrogen, 15596018). The concentration of the RNA was qualified with NanoDrop 2000 (ThermoFisher Scientific, USA). Complementary DNA was prepared from 1 μg of mRNA using an Evo M-MLV reverse transcription II kit (Accurate Biology, AG11711) following the manufacturer’s protocol. Gene expression analysis was performed on a LightCycler 96 instrument (Roche, Switzerland) with PerfectStart Green qPCR SuperMix (TransGen Biotech, AQ602–02). QPCR primer sequences are provided in Table S3.

### Animal Models and Designs

All animal research procedures we conducted (Approval Number: GY2022–045) were approved by the Institutional Animal Care and Use Committee at Guangzhou Medical University. Male C57BL/6J mice (10–12 weeks old, weighing 25 ± 2 g, Spfbiotech, Beijing, China) were maintained under standard housing conditions. On day 0, mice were randomly assigned to receive either a single dose of bleomycin (BLM, 1.5 U/kg, Hanhui Pharmaceuticals Co., Ltd., Hangzhou, China) or an equal volume (50 μL) of sterile 0.9% saline via intratracheal instillation.^34^ To determine the dose–response preventive effects of CQD in BLM-induced pulmonary fibrosis, mice were intraperitoneally administered three different doses of CQD (2, 4, and 8 mg/kg) every other day from day 1 to day 20. The control and bleomycin groups were treated with a vehicle using the same procedure. Pirfenidone (150 mg/kg, MACKLIN, M823668) was administered intragastrically as the positive control. Pulmonary function was monitored weekly using unrestrained whole-body plethysmography (EMKA, Germany). Body weights were measured every other day to monitor mice growth and survival. End points included death and weight loss of 27% or more of body weight, resulting in euthanasia of all mice. On day 21, lung tissues, bronchoalveolar lavage fluid (BALF), and serum were collected for further analysis. Additionally, hearts, livers, and other organs were collected and fixed in 10% paraformaldehyde for organ toxicity assessment. For the therapeutic assessment, drug intervention in pulmonary fibrosis mice began on day 9 after the bleomycin challenge with a repeated dosing schedule (once every 2 days for six treatments over 13 days). The mice were randomly assigned to the following treatment groups: 1) Control group (Ctrl); 2) BLM group; 3) BLM+ CQD group (2 mg/kg, i.p); 4) BLM+ CQD group (4 mg/kg, i.p); 5) BLM+ CQD group (8 mg/kg, i.p); 6) Nintedanib treatment group, where mice received nintedanib (60 mg/kg, i.g, MACKLIN, N856623). Lung structure and aeration were assessed using microcomputed tomography (Micro-CT) on day 21, after which the mice were euthanized, and samples were collected as described earlier.

### Pulmonary Function Measurement

Lung function was noninvasively evaluated using an EMKA whole-body plethysmography system. Mice were placed comfortably in the plethysmography chambers and allowed a 20 min acclimatization period before data collection began. Measurements were taken after device calibration, and respiratory parameters, such as midexpiratory tidal flow (EF_50_) and Penh, were calculated using iox2 software.

### *In Vivo* Micro-CT Imaging

The mice were fasted for 12 h before anesthesia but allowed free access to water. After anesthesia with 2% isoflurane, the mice were scanned with Quantum GX Micro-CT (PerkinElmer, USA). The images were acquired using the following parameters: X-ray tube voltage of 90 kV, X-ray tube current of 88 μA, total scan time of 4 min, with an X-ray filter of 0.5 mm Aluminum (Al) and 0.06 mm Copper (Cu). Postprocessing was performed using Analyze software (Analyze 14.0). Briefly, a semiautomatic lung segmentation algorithm was used to quantity pulmonary parenchymal lesions. Lung regions were identified into three classifications by CT attenuation densities:1) normally aerated, with a density between −860 and −435 Hounsfield units (HU); 2) poorly aerated, with a density between −434 and −121 HU; 3) nonaerated, with a density between −120 and +121 HU.^23,24^

### Measurement of Hydroxyproline Levels

The hydroxyproline contents in the lungs were examined using a commercial kit (Nanjing Jiancheng Bioengineering Institute, A030–2–1, China) in compliance with the product specification. Lung tissues were weighed, hydrolyzed in a basic solution, pH adjusted, and then mixed with the detection buffer. The supernatant was collected and read at 550 nm after incubation in a water bath, utilizing a Synergy H1 microplate reader (BioTek, USA).

### HE and Masson’s Trichrome Staining

The tissues were rinsed with a saline solution and then fixed in 10% paraformaldehyde overnight. Following dehydration through an ethanol series, the tissues were embedded in paraffin to cut 5 μm tissue sections according to standard laboratory procedures. Then, the tissue sections were deparaffinated and rehydrated in xylene and different ethanol concentrations. Subsequently, HE and Masson’s trichrome staining were performed according to the kit’s instructions (Solarbio, Beijing, China), and histopathological changes were observed under the Aperio CS2 whole slides scanner (Leica, Germany). HALO software was applied to calculate collagen areas.

### Cytokine Measurements by ELISA

The BALF and serum samples were separated using previously described procedures.^51^ The levels of murine IL-6, oncostatin M, IL-1β, and TGFβ1 in BALF or serum were quantified using the following ELISA kits according to the manufacturers’ instructions: IL-6 Mouse uncoated EL kit (Invitrogen, 88–7064–88), IL-1β Mouse uncoated ELISA kit (Invitrogen, 88–7013A-88), TGF β-1 Human/Mouse Uncoated ELISA Kit (Invitrogen, 88–8350–88), and mouse Oncostatin M (OSM) ELISA Kit (Cloud Clone, SEA110Mu).

### Immunofluorescence Staining

For the paraffin-embedded lung tissue, the sections were dewaxed, rehydrated, and underwent antigen retrieval before being incubated with the following primary antibodies: Collagen I (Abcam, ab34710, 1:500) and α-SMA (Servicebio, GB13044–50, 1:1000). The Slides were then washed three times in 0.02% Triton X-100 in PBS and incubated with 488 and 594 Alexa Fluor secondary antibodies (1:400, Invitrogen, A21207, A28175) for 1 h in the dark, followed by DAPI staining for 5 min. Fluorescent imaging was acquired with a digital slide scanner system (3D HISTECH, Hungary) and analyzed by HALO software.

### DARTS Assay

A DARTS assay was performed with modifications to a previously published method to identify potential binding targets of CQD.^52^ NIH/3T3 cells were stimulated with 10 ng/mL TGFβ1 for 48 h. Subsequently, the collected cells were lysed with M-PER mammalian protein extraction reagent (ThermoFisher Scientific, 78503) containing Halt protease and phosphatase inhibitor cocktail (ThermoFisher Scientific, 1861280) and centrifuged at 13,000 rpm for 15 min at 4 °C. The supernatant was carefully aspirated, and the cell pellet was then resuspended in TNC buffer (50 mM Tris-HCl, 50 mM NaCl, and 10 mM CaCl_2_). Following protein quantification, the protein was incubated with CQD or DMSO at room temperature for 30 min on a rotator, followed by digestion with Pronase (Roche, 10165921001) at room temperature for 20 min in the appropriate ratio. The reaction was stopped by adding a protease inhibitor cocktail on ice for 10 min. Samples were heated in an SDS loading buffer for Western blot analysis or silver staining. The protected gel bands were cut out and underwent in-gel digestion procedures for further liquid chromatography and mass spectrometry (LC/MS) analysis.

### Quantitive Proteomic Analysis by LC/MS

Peptides extracted from the DARTS assay were analyzed using Orbitrap Fusion Lumos (ThermoFisher Scientific) coupled with an EASY-nLC 1000 ultrahigh-pressure liquid chromatography system (ThermoFisher Scientific). Then the MS data were processed with the SEQUEST HT search algorithm in Proteome Discoverer 2.4 software (ThermoFisher Scientific) against the Swissprot database. The processed data was transferred to the R software for statistical analysis and visualization. The criteria for identifying potential hits were defined as 1) fold change greater than 1.2 or less than 0.8333; 2) p-value less than 0.05.

### SPR Analysis

To confirm the potential binding targets of CQD, 10 μM CQD in DMSO and control samples were immobilized on a three-dimensional Photo-cross-linker Sensor CHIP (Betterways Inc., China) using the AD 1520 Array Printer (Bidot Inc., USA) through C–H covalent bonds. Following that, different dilutions of purified recombinant proteins were passed through the chip at a rate of 0.5 μL/s in PBST (pH 7.4, with 0.1% Tween 20) for 600 s at 4 °C. The proteins were eluted from the chip at a flow rate of 2 μL/s in a regeneration buffer (10 mM glycine-HCl, pH 2.0) at 4 °C for 360 s. The response units (RU) were measured using the bScreen LB 991 Label-free Microarray System (Berthold Technologies, Germany). The collected data were analyzed using the Langmuir binding model by the system. To assess the affinity between MTRR subdomains and CQD, peptides 1, 2, and 3 were synthesized by Genscript (Wuhan, China) following molecular docking. Subsequently, SPR assays were conducted following the aforementioned procedures.

### CETSA

Stimulated NIH/3T3 cells were collected, resuspended in PBS, and aliquoted in equivalent volumes. For the temperature range experiment, aliquots were treated with chlorquinaldol (100 μM) or DMSO for 30 min at room temperature. Whereafter, samples were divided into 100 μL/tube and heated at desired temperatures for 3 min. Following three freeze–thaw cycles, soluble proteins were separated for Western blot. To assess isothermal concentration response, collected cells were dosed with a dilution series of chlorquinaldol concentrations at room temperature for 30 min, denatured for 3 min at 52 °C, and isolated soluble proteins for analysis.

### Molecular Docking

To model the mouse methionine synthase reductase (MTRR), we obtained the protein sequence from UniProt (Q83C1A3) and used the human MTRR template file from the Protein Data Bank (PDB ID: 2QTZ). Next, we utilized the SWISS-MODEL online platform to generate a homology model of the mouse MTRR, which was subsequently optimized using the Protein Preparation Wizard Maestro software package (Schrödinger, Inc., USA). For the ligand, the 3D structure of CQD was processed using the LigPrep module (Schrödinger, Inc., USA). Subsequently, the prepared CQD was docked into the grid box using the extra precision (XP) mode of the Glide program from the Schrödinger suite. The docking parameters were set to extra precision (XP), with flexible ligand sampling and 10 poses per ligand. All other parameters remained at their default values. The docked complexes were calculated using the Prime Molecular Mechanics Generalized Born Surface Area (MMGBSA) tool. Finally, the docking pose with the top three lowest MMGBSA scores was visualized using PyMOL.

### Molecular Dynamics Simulation

The molecular dynamics (MD) simulations were conducted using the Desmond software package from D.E. Shaw Research, Schrödinger, Inc., USA. The docked complex was solvated using the single-point charge (SPC) water model, and a physiological concentration of 0.15 M NaCl was added for simulation. Additional Na^+^/Cl^–^ ions were added as needed to maintain electrical neutrality. Subsequently, the solvated system underwent minimization with 1000 steps using standard Desmond protocols before initiating a 20 ns MD simulation. The simulation was set up in the NPT ensemble with the OPLS4 force field to keep the system at 300 K temperature and 1-atm pressure. For analyzing the interactions between CQD and MTRR, the Schrödinger Simulation Interactions Diagram (SID) tool was used. Additionally, the root-mean-square deviation (RMSD) of the ligand-protein complex was computed to assess the stability of the ligand binding during the simulation.

### Determination of cellular SAM, SAH, and Methionine Levels by HPLC/MS Assay

The Internal standard solution of l-phenylalanine-d5 (MedChemExpress, HY-N0215S12) was dissolved in 50% methanol–water at a concentration of 200 ng/mL. To extract intracellular metabolites, 5 × 10^6^ cell samples were scraped off plates into prechilled PBS, centrifuged, resuspended in 75% methanol–water (Merck, 106035), sonicated for 5 min in an ice bath, and then centrifuged for 15 min at 17,000 g. Subsequently, either the supernatant or the standard solution [S-adenosylmethionine (SAM, Selleck, S5109), S-adenosyl-l-homocysteine (SAH, Selleck, S7868), or l-methionine (Solarbio, M0010)] was mixed with the equal amount of internal standard l-phenylalanine-d5. Then, the mixture was separated by ACQUITY ultrahigh-performance liquid chromatography (Waters, USA) with clipse Plus C18 RRHD column (1.8 μm, 2.1 × 50 mm, Agilent Technologies, USA). For positive mode analysis, the initial mobile phase consisted of 95% 0.1% formic acid, and 5% acetonitrile at 0.4 mL/min for the first minute. After 1 min, the gradient from 5% to 95% acetonitrile was run at 0.4 mL/min within 2.5 min, followed by 95% acetonitrile for 0.5 min. The column was then equilibrated for 1 min to the starting conditions. Subsequently, the indicated metabolites were monitored using an ABSciex 4500 Triple Quadrupole Mass Spectrometer (ABSciex, USA) instrument operating with an electrospray ionization source (ESI). The mass spectrometer was run in the Multiple Reaction Monitoring (MEM) mode with the interface heated to 500 °C. The capillary voltage was set to 4.5 kV, with the collision-activated dissociation (CAD) gas pressure at 10 psi and the curtain gas pressure at 25 psi. The following ion transitions were used for quantification: SAM (*m*/*z* 399.1 → 250.1), SAH (*m*/*z* 385.1 →136.1), methionine (*m*/*z* 150.1 → 56.2), and l-phenylalanine-d5 internal standard (*m*/*z* 171.1 →124.6). Data analysis was carried out by MultiQuant 3.0.3 software (ABSciex, USA).

### Knockdown of *Mtrr* by shRNA

The short hairpin RNA targeting *Mtrr* (shMtrr) and scramble plasmid were bought and cloned into the GV493 vector (Shanghai GeneChem Co., Ltd., China). The sequences for mouse Mtrr shRNA and scramble were provided in Table S4. To package the inserted vector into lentivirus, 293T cells were cotransfected with the GV493 vector, pHepler 2.0, and pHepler 1.0 (Shanghai GeneChem Co., Ltd., China). Following packaging, we transfected the lentivirus into NIH/3T3 cells at a MOI of 100 for 8 h. After 48 h, 4 μg/mL puromycin was added to select the stably downregulated Mtrr cells. The efficiency of lentivirus infection was examined by observing the green signal under an EVOS microscope (ThermoFisher Scientific, USA) and reverse transcription-quantitative PCR.

### MTRR Activity Detection

Kinetic assays were conducted in a 96-well plate at 37 °C, with absorbance changes at 550 nm being monitored to track the reduction of cytochrome c. The reaction mixtures consisted of 30 mM potassium phosphate buffer (pH 7.8), 144 μM cytochrome c (Sigma, C2506), 74.4 μM NADPH (Topscience, T19467), and 32 mg of protein samples.

### Statistic and Analysis

The data presented here show the mean values along with the standard error of the mean (SEM), and all experiments were independently conducted at least three times. Statistical analysis was performed using GraphPad Prism 8.0 software. To assess statistical significance, either a two-tailed *t* test or-way analysis of variance (ANOVA) followed by a Bonferroni-corrected *t* test was performed. A p-value of less than 0.05 was considered statistically significant.

## Supporting Information Available

Effects of chlorquinaldol treatment, bioinformatics and SPR analysis of chlorquinaldol’s candidate targets, molecular dynamics analysis, myofibroblast differentiation and fibroblast activation analysis, pharmacokinetic parameters of CQD, small molecule compounds used in the screening experiment, potential target compounds of clorquinaldol, PCR primer sequences, and ShRNA target sequences (PDF)

## Abbreviations

α-SMA: α-smooth muscle actin
BLM: bleomycin
CETSA: cell thermal shift assay
CQD: chlorquinaldol
DARTS: drug affinity responsive target stability assay
ECM: extracellular matrix
ELISA: enzyme-linked immunosorbent assay
FMT: fibroblast-to-myofibroblast transition
GARS1: glycyl-tRNA Synthetase 1
HE: hematoxylin and eosin
INPPL1: inositol polyphosphate-specific phosphatase 1
IPF: idiopathic pulmonary fibrosis
Ka: association rate constant
*K*_d_: dissociation rate constant
K_D_: equilibrium dissociation constant
MD: molecular dynamics
MTRR: methionine synthase reductase
OSM: oncostatin M
SPR: surface plasmon resonance
SAH: *s*-adenosylhomocysteine
SAM: *s*-adenosylmethionine
