# Supplementary material for: Chlorquinaldol Alleviates Lung Fibrosis in Mice by Inhibiting Fibroblast Activation through Targeting Methionine Synthase Reductase
Source: ACS Cent Sci. 2024 Aug 28;10(9):1789–802. doi: 10.1021/acscentsci.4c00798 (PMC11428390; doi:10.1021/acscentsci.4c00798)
Supplement: Supplementary file 1 — oc4c00798_si_001.pdf [file oc4c00798_si_001.pdf]

# Chlorquinaldol Alleviates Lung Fibrosis in Mice Through Inhibiting Fibroblast Activation by Targeting Methionine Synthase Reductase

Xiangyu Yang,† Geng Lin,† Yitong Chen,† Xueping Lei,† Yitao Ou,Yuyun Yan, Ruiwen Wu, Jie Yang, Yiming Luo, Lixin Zhao, Xiuxiu Zhang, Zhongjin Yang, Aiping Qin, Ping Sun*, Xi-Yong Yu*, Wenhui Hu*

The Fifth Affiliated Hospital, Guangzhou Municipal and Guangdong Provincial Key Laboratory of Molecular Target & Clinical Pharmacology, the NMPA and State Key Laboratory of Respiratory Disease, School of Pharmaceutical Sciences, Guangzhou Medical University, Guangzhou, 511436, China

* Correspondence

E-mail: sun_ping@gzhmu.edu.cn

E-mail: yuxycn@gzhmu.edu.cn

E-mail: huwenhui@gzhmu.edu.cn

†All these authors contributed equally to this work.

This file includes:

Supplementary Materials

Figure S1. Inhibition of Extracellular Matrix Remodeling by Chlorquinaldol Treatment.

Figure S2. Chlorquinaldol Reduces Inflammation and Presents a Favorable Safety Profile.

Figure S3. Bioinformatics and SPR Analysis of Chlorquinaldol's Candidate Targets.

Figure S4. Molecular Dynamics Analysis of Chlorquinaldol Binding to MTRR.

Figure S5. Chlorquinaldol Exhibits No Impact on MTRR Transcription and Translation.

Figure S6. Chlorquinaldol Enhances MTRR Activity.

Figure S7. Myofibroblast Differentiation is Folic Acid-Independent.

Figure S8. The Methionine Metabolism Pathway Influences Fibroblast Activation.

Table S1. Small Molecule Compounds were Used in the Screening Experiment.

Table S2. Pharmacokinetic Parameters of CQD after a Single Dose in C57BL/6J mice.

Table S3. The List of 78 Potential Target Proteins of Chlorquinaldol.

Table S4. PCR Primer Sequences.

Table S5. ShRNA target Sequences.

# Table of Contents for Supporting Information

1. Figures S1-8 .......... S3

2. Tables S1-5 .......... S8

## 1. Figures S1-8

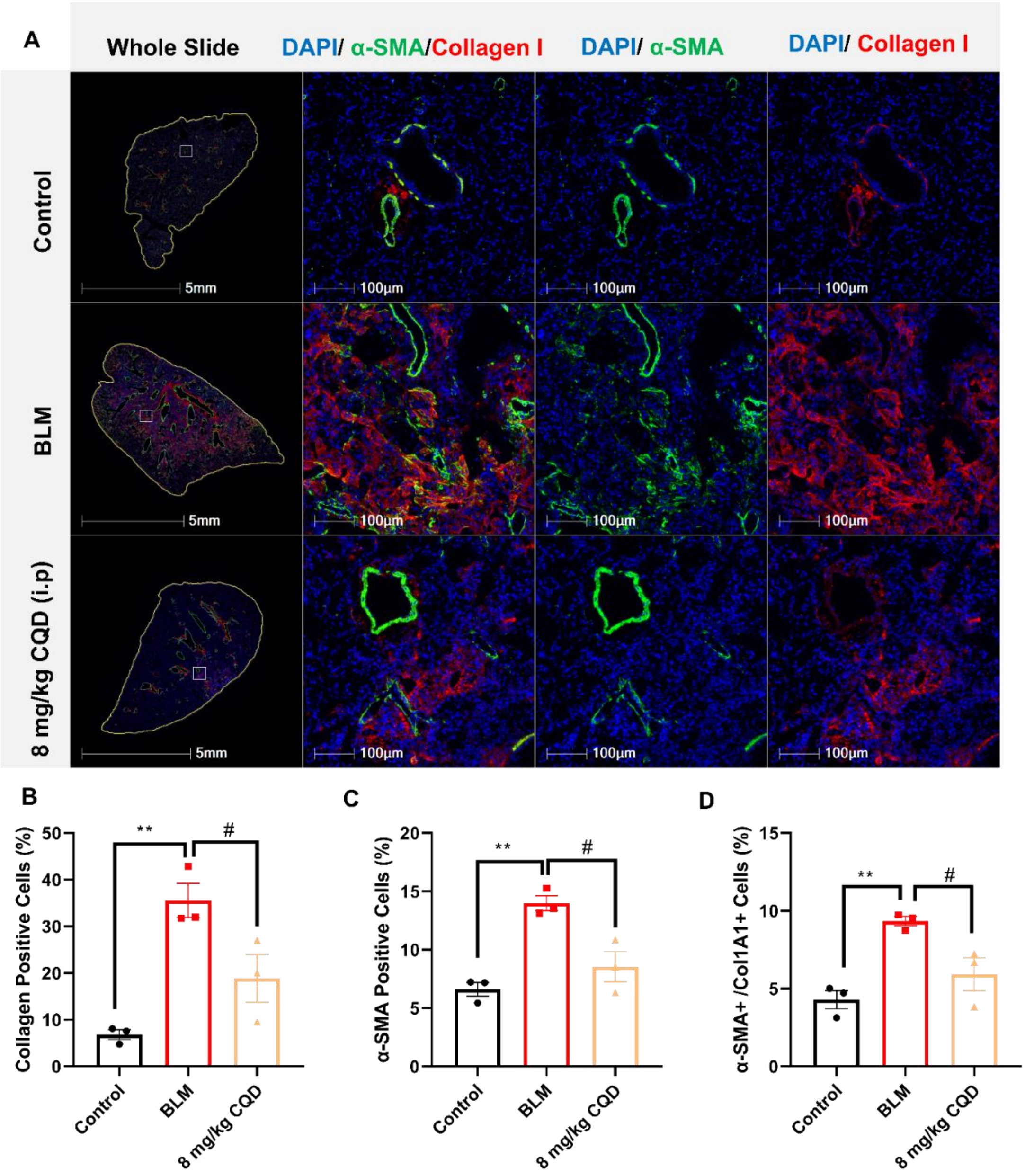


**Figure S1. Inhibition of extracellular matrix remodeling by chlorquinaldol treatment.** A: Representative images of Immunofluorescence staining with α-SMA and Collagen I in lung sections. Red, Collagen I; green, α-SMA; blue, DAPI. B-D: Quantification data of Collagen I positive cells., α-SMA positive cells, and α-SMA$^{+}$/ Collagen I$^{+}$ cells. Data is expressed as mean ± SEM (n=3). Significance is denoted by $^{*}$p<0.05, $^{**}$p<0.01, compared to control; $^{\#}$p<0.05 for comparisons with the BLM group.

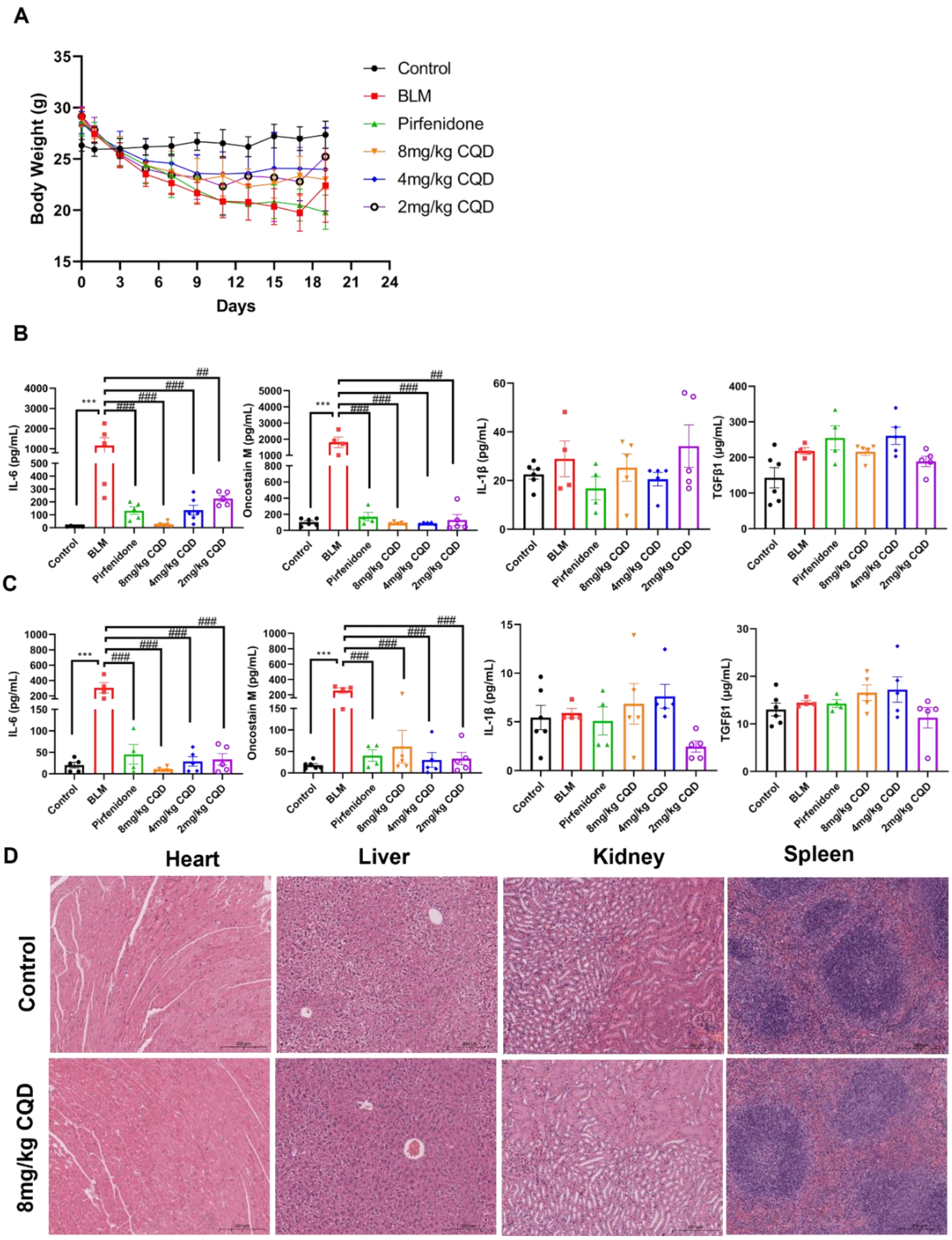


**Figure S2. Chlorquinaldol reduces inflammation and presents a favorable safety profile.** A: Body weight changes in mice over 21 days (n=6-10). B, C: The levels of IL-6, OSM, IL-1β, and TGFβ1 in bronchoalveolar lavage fluid (BALF) and serum for each experimental group. Data is expressed as mean ± SEM, with n=4-6 per group. $^{*}p<0.05$, $^{**}p<0.01$, $^{***}p<0.001$ versus the control group; $^{\#}p<0.05$, $^{\#\#}p<0.01$, $^{\#\#\#}p<0.001$ versus the BLM group. D: Evaluate the safety of CQD on cardiac, hepatic, and splenic tissues through hematoxylin and eosin (HE) staining.

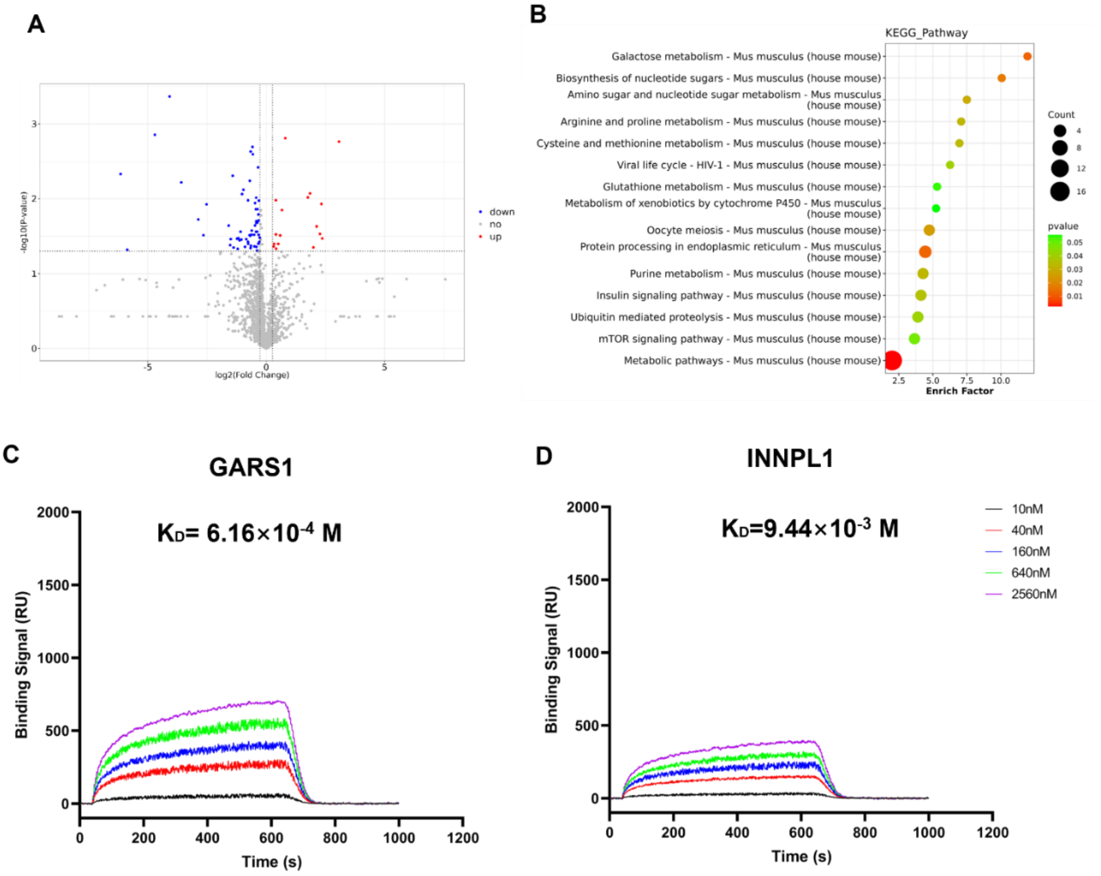


**Figure S3. Bioinformatics and SPR analysis of chlorquinaldol's candidate targets.** A: Volcano plot illustrates proteins with significant differences between the control and CQD-treated groups in the DARTS/MS assay. Proteins are categorized by their -$\log_{10}$(p-values) on the y-axis and $\log_2$(fold change) on the x-axis, with upregulated proteins in red, downregulated in blue, and non-significant in gray. B: KEGG pathway analysis reveals the functional properties of potential CQD targets, visualized as a bubble chart of the top 15 enriched pathways. C: SPR binding curves for CQD interaction with recombinant GARS1 protein at concentrations ranging from 10 nM to 2560 nM. D: SPR binding profiles for CQD with recombinant INPPL1 protein over the same concentration range as in panel C.

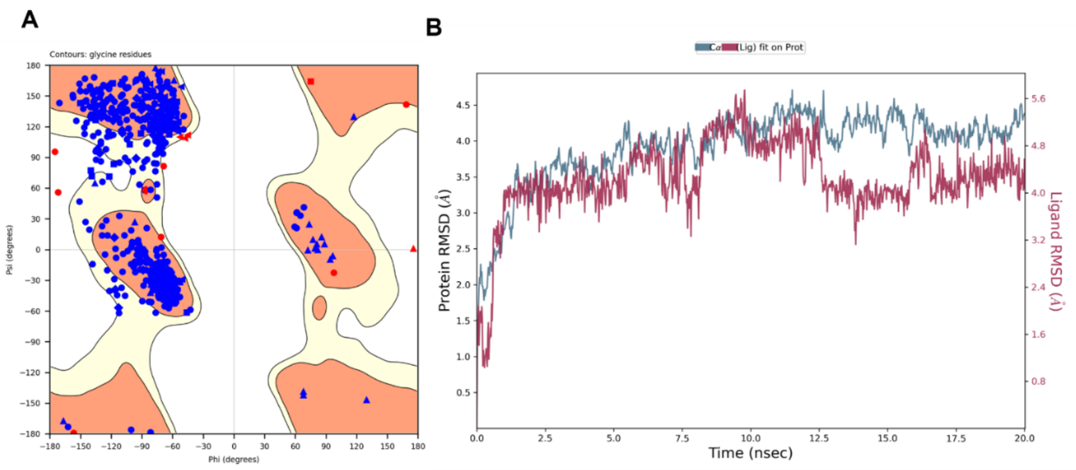


**Figure S4. Molecular dynamics analysis of chlorquinaldol binding to MTRR.** A: Features the Ramachandran Plot for the optimized mouse MTRR homology model, with each point corresponding to an amino acid. Amino acid stability is denoted by a color gradient from red (most stable) to yellow to white (least stable). The model indicates that 90.35% of residues are in the acceptable region, 8.85% in the additional allowed region, and 0.8% in the generously allowed region. B: The RMSD plot for the Chlorquinaldol - MTRR complex throughout the molecular dynamics simulation, providing insight into the complex's structural stability over time.

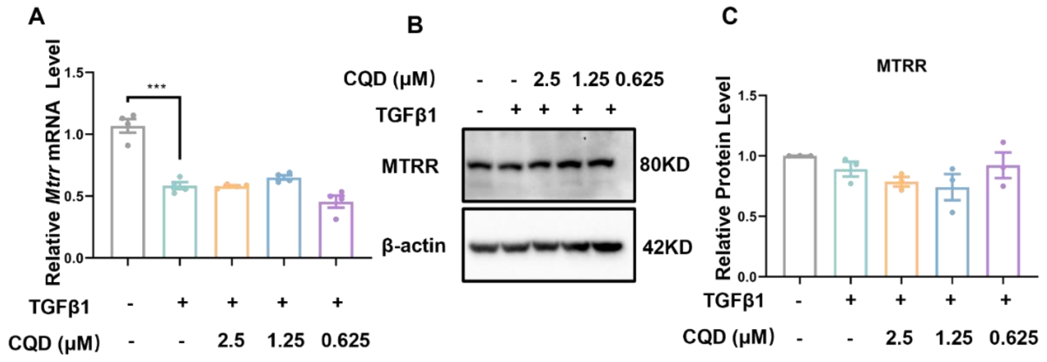


**Figure S5. Chlorquinaldol exhibits no impact on MTRR transcription and translation.** A: qPCR assessment of *Mtrr* mRNA levels following exposure to different Chlorquinaldol (CQD) concentrations (n=4). Data are expressed as mean ± SEM. B, C: Western blot determination of MTRR protein levels in response to different CQD concentrations (n=3). Results are depicted as mean ± SEM. Statistical significance is indicated by $^{*}$p<0.05, $^{**}$p<0.01, and $^{***}$p<0.001 for

comparisons against the control group.

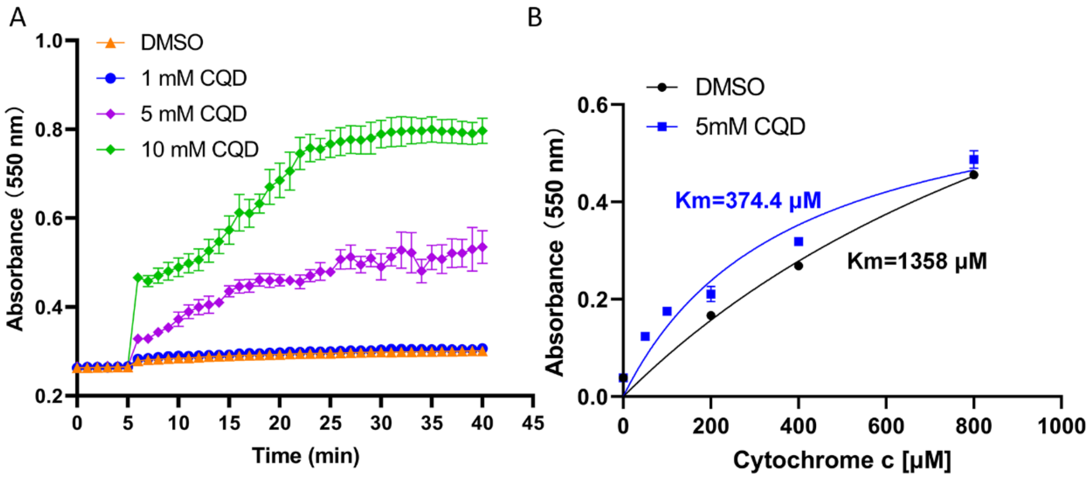


**Figure S6. Chlorquinaldol enhances MTRR activity.** A. A mixture of 144 μM cytochrome c and 74.4 μM NADPH was incubated at 37°C in the assay buffer and monitored by absorption at 550 nm for 5 minutes. The addition of protein samples and different concentrations of chlorquinaldol (CQD) induced cytochrome c reduction, and the rate of product formation increased with CQD concentration (n=4). B. In substrate specificity tests with varying cytochrome c levels over 30 minutes, the Km values were 1358 μM without CQD and 374.4 μM with CQD (n=3).

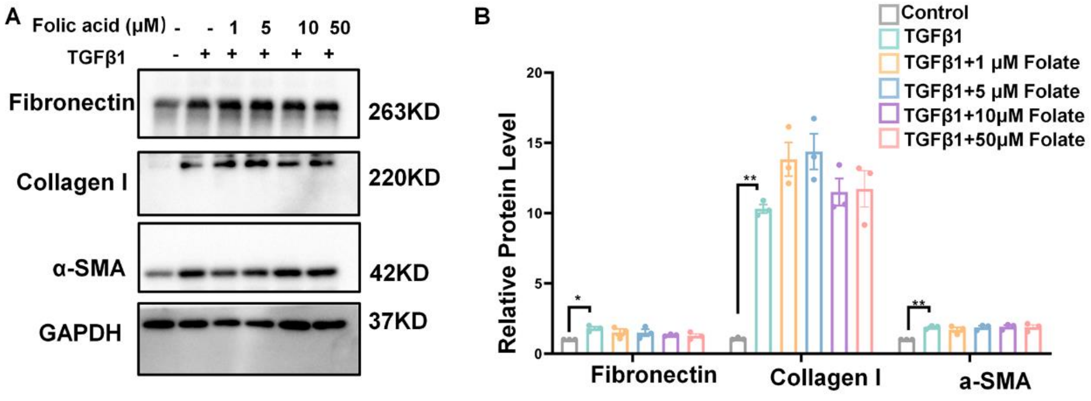


**Figure S7. Myofibroblast differentiation is folic acid-independent.** A, B: Following 48-hour TGFβ1 exposure, NIH/3T3 fibroblasts were treated with varying folic acid concentrations for 24 hours. Protein levels of fibronectin, collagen I, and α-SMA were assessed using western blot. Data is displayed as mean ± SEM (n=3). $^{*}$p<0.05, $^{**}$p<0.01, $^{***}$p<0.001 signify statistical significance compared to the control group. $^{\#}$p<0.05, $^{\#\#}$p<0.01, and $^{\#\#\#}$p<0.001 indicate statistical significance when compared to the TGFβ1-treated group.

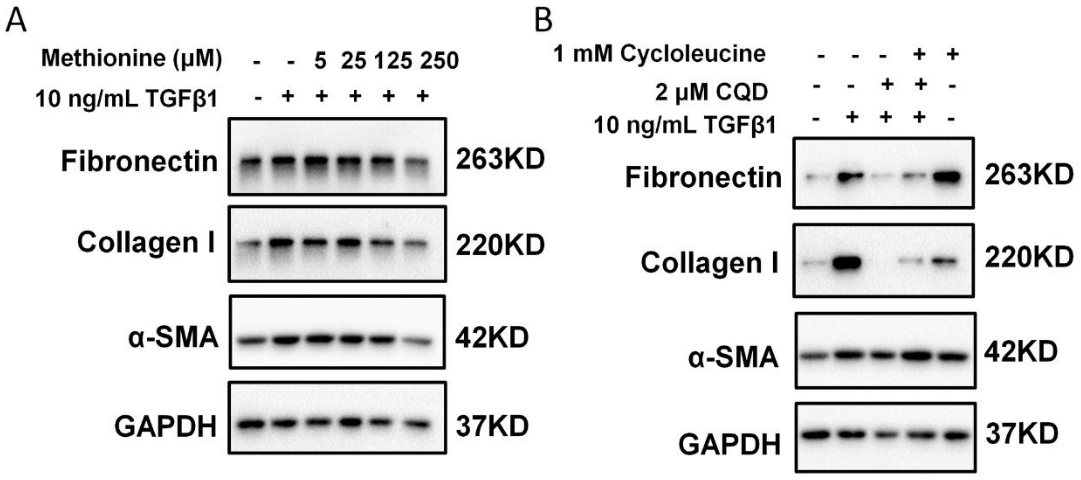


**Figure S8. The methionine metabolism pathway influences fibroblast activation.** A. NIH/3T3 cells are pre-treated with different concentrations of methionine for 24 hours prior to TGFβ1 stimulation, and western blot analysis is performed to evaluate the expression levels of fibronectin, collagen I, and α-SMA. B. After a 24-hour treatment with CQD and/or Cycloleucine, NIH/3T3 cells underwent western blot analysis to measure the protein expression levels of fibronectin, collagen I, and α-SMA, using GAPDH as the loading control.

## 2. Tables S1-5

**Table S1. Small molecule compounds were used in the screening experiment.**

| Vial | MCE Cat.No | Compound name |
|---|---|---|
| 1 | HY-50904 | Nintedanib |
| 2 | HY-B1304 | (-)-Sparteine (sulfate pentahydrate) |
| 3 | HY-B0223 | Albendazole |
| 4 | HY-B0509B | Amikacin (sulfate) |
| 5 | HY-B0771A | Cefozopran (hydrochloride) |
| 6 | UY-17459 | Clopidogrel (hydrogen sulfate) |
| 7 | HY-B0382 | Fosinopril (sodium) |
| 8 | HY-B0003 | Gemcitabine (Hydrochloride) |

| | | |
|---|---|---|
| 9 | HY-B0912B | Piperazine (malate) |
| 10 | HY-13757 | Tamoxifen (Citrate) |
| 11 | HY-17387 | (-)-Huperzine A |
| 12 | HY-B0471 | (R)-(-)-Phenylephrine (hydrochloride) |
| 13 | HY-B0891 | 17-Hydroxyprogesterone |
| 14 | HY-B1428 | 2-Ethoxybenzamide |
| 15 | HY-B1140 | 3-Methyl-7-chloro-1,2,4-benzothiadiazir |
| 16 | HY-B1398 | 4-Aminoantipyrine |
| 17 | HY-N0305 | 5-Aminolevulinic acid (hydrochloride) |
| 18 | HY-15531 | ABT-199 |
| 19 | HY-B1510 | Acrivastine |
| 20 | HY-B0379A | Adiphenine (hydrochloride) |
| 21 | HY-B0107 | Adtretin |
| 22 | HY-B0192 | Alfuzosin |
| 23 | HY-B0500 | Alverine (citrate) |
| 24 | HY-13209 | Ambnsenian |
| 25 | HY-B0713 | Amlexanox |
| 26 | HY-B0467B | Amoxicillin (trihydrate) |
| 27 | HY-17430 | Amprenavir |
| 28 | HY-14274 | Anastrozole |
| 29 | HY-50667 | Apixaban |
| 30 | HY-NO192 | Arbutin |

| | | |
|---|---|---|
| 31 | HY-N0034 | Arctiin |
| 32 | HY-N0402 | Artemether |
| 33 | HY-B0094 | Artemisinin |
| 34 | HY-13557 | Ascomycin |
| 35 | HY-18252 | Avanafil |
| 36 | HY-A0022A | Azaphen dihydrochloride monohydrate |
| 37 | HY-N0197 | Baicalin |
| 38 | HY-15315A | Baricitinib (phosphate) |
| 39 | HY-B0892 | Benzyl alcohol |
| 40 | HY-B0134 | Bestatin |
| 41 | HY-B0301 | Bifonazole |
| 42 | HY-B0511 | Biotin |
| 43 | HY-13578 | Brivudine |
| 44 | HY-12015 | BSI-201 |
| 45 | HY-B0764 | Bucladesine sodium |
| 46 | HY-B0494 | Bufexamac |
| 47 | HY-17468 | Bumetanide |
| 48 | HY-17573 | Carbetocin |
| 49 | HY-B0558 | Carbimazole |
| 50 | HY-13585 | Carmustine |
| 51 | HY-B0006 | Carvedilol |
| 52 | HY-B1257 | Cefmetazole (sodium) |

| | | |
|---|---|---|
| 53 | HY-B0458 | Cefprozil (monohydrate) |
| 54 | HY-B0593 | Ceftazidime |
| 55 | HY-B0698A | Ceftibuten dihydrate |
| 56 | HY-12048 | Chelerythrine Chloride |
| 57 | HY-76847 | Chenodeoxycholic Acid |
| 58 | HY-13593 | Chlorambucil |
| 59 | HY-80608 | Chlorhexidine (digluconate) |
| 60 | HY-B0911 | Chlorophyllin (sodium copper salt) |
| 61 | HY-B0224 | Chlorothiazide |
| 62 | HY-B0286A | Chlorpheniramine (maleate) |
| 63 | HY-B0274 | Chlorprothixene |
| 64 | HY-B1360 | Chlorquinaldol |
| 65 | HY-14739 | Choline Fenofibrate |
| 66 | HY-B0408A | Clindamycin hydrochloride |
| 67 | HY-A0005 | Clofarabine |
| 68 | HY-B1046 | Clofazimine |
| 69 | HY-B0463 | Clomiphene (citrate) |
| 70 | HY-B1165 | Cyproheptadine hydrochloride sesquihydrate |
| 71 | HY-17560 | Demeclocycline (hydrochloride) |
| 72 | HY-B0602A | Des venlafaxine (succinate hydrate) |
| 73 | HY-B0552A | Di bucaine (hydrochloride) |
| 74 | HY-15037 | Diclofenac (Sodium) |
| 75 | HY-N0176 | Dihydroartemisinin |
| 76 | HY-14656 | Diltiazem (hydrochloride) |
| 77 | HY-B1022 | Dimesna |
| 78 | HY-17363 | Dimethyl fumarate |

| | | |
|---|---|---|
| 79 | HY-A0082 | Diphenidol (hydrochloride) |
| 80 | HY-14393 | Emodin |
| 81 | HY-17427 | Emtricitabine |
| 82 | HY-13624A | Epirubicin (hydrochloride) |
| 83 | HY-B0234 | Estrone |
| 84 | HY-B1361 | Estropipate |
| 85 | HY-17376 | Ezetimibe |
| 86 | HY-17426 | Famciclovir |
| 87 | HY-16637 | Folic acid |
| 88 | HY-N0382 | Galangin |
| 89 | HY-13637 | Ganciclovir |
| 90 | HY-10581 | Gatifloxacin |
| 91 | HY-N0010 | Geniposidic acid |
| 92 | HY-B0355 | Ginkgolide A |
| 93 | HY-N0039 | Ginsenoside Rb1 |
| 94 | HY-B0071A | Granisetron (Hydrochloride) |
| 95 | HY-B0178A | Guanidine hydrochloride |
| 96 | HY-N0167 | Gynostemma Extract |
| 97 | HY-B0547A | Homatropine Bromide |
| 98 | HY-B0547A | Homatropine Bromide |
| 99 | HY-17009 | Iguratimocl |
| 100 | HY-B1451 | Imidapril (hydrochloride) |
| 101 | HY-N0170 | Indole-3-carbinol |
| 102 | HY-12222 | INT-747 |
| 103 | HY-B1469 | Isosorbide |
| 104 | HY-B0642 | Isosorbide mononitrate |
| 105 | HY-15127 | Isotretinoin |
| 106 | HY-N0659 | Jujuboside A |
| 107 | HY-16566A | Kanamycin (sulfate) |

| | | |
|---|---|---|
| 108 | HY-B0347 | Lacidipine |
| 109 | HY-B1030 | Lanatoside C |
| 110 | HY-50898A | Lapatinib (ditosylate) |
| 111 | HY-B1071 | Lasalocid |
| 112 | HY-A0038 | Lasofoxifene tartrate |
| 113 | HY-15777 | LEE011 |
| 114 | HY-15777B | LEE011 (succinate) |
| 115 | HY-15258A | Lesinurad sodium |
| 116 | HY-14248 | Letrozole |
| 117 | HY-B0381B | Levobetaxolol (hydrochloride) |
| 118 | HY-14286 | Levosimendan |
| 119 | HY-B0185A | Lidocaine (hydrochloride) |
| 120 | HY-B1358 | Lincomvcin (hydrochloride hydrate) |
| 121 | HY-B0455 | Lomefloxacin (hydrochloride) |
| 122 | HY-B0517A | Mepivacaine (hydrochloride) |
| 123 | HY-B0209 | Metolazone |
| 124 | HY-B0454A | Miconazole nitrate |
| 125 | HY-B0884 | Minaprine |
| 126 | HY-B1484 | Moxalactam (sodium salt) |
| 127 | HY-B0199 | Mycophenolate Mofetil |
| 128 | HY-B0506 | Nadifloxacin |
| 129 | HY-B0555A | Nafcillin (sodium monohydrate) |
| 130 | HY-B1057 | Nefopam (hydrochloride) |
| 131 | HY-17357 | Nepafenac |
| 132 | HY-B0341 | Nicorandil |
| 133 | HY-14284 | Nilvadipine |
| 134 | HY-17402 | Nisoldipine |
| 135 | HY-B0424 | Nitrendipine |
| 136 | HY-B0528A | Octopamine (hydrochloride) |

| | | |
|---|---|---|
| 137 | HY-14254 | Olprinone (Hydrochloride) |
| 138 | HY-A0019 | Paliperidone |
| 139 | HY-17474 | Parecoxib |
| 140 | HY-17474A | Parecoxib (Sodiu) |
| 141 | HY-B0724A | Pazufloxacin mesylate |
| 142 | HY-10997 | PCI-32765 |
| 143 | HY-13781 | Pemetrexed (disodium hernipenta hydate) |
| 144 | HY-B0715 | Pentoxifylline |
| 145 | HY-B0887 | Permethrin |
| 146 | HY-B0967 | Phthalylsulfacetamide |
| 147 | HY-13723 | Pimecrolimus |
| 148 | HY-B1210 | Pipemidic acid |
| 149 | NY-17037 | Pirenzepine (dihydrochloride) |
| 150 | HY-B0253 | Piroxicam |
| 151 | HY-50912 | Plerixafor (octahydrochloride) |
| 152 | HY-N0120A | Polydatin |
| 153 | HY-12047 | Ponatinib |
| 154 | HY-17455 | Pramiracetam |
| 155 | HY-B1319 | Pramocaine (hydrochloride) |
| 156 | HY-B0339 | Primidone |
| 157 | HY-B0306 | prothionamide |
| 158 | HY-B0477 | Quinapril hydrochloride |
| 159 | HY-B0269 | Rifapentine |
| 160 | HY-15455 | Roflumilast |
| 161 | HY-14600 | Rosiglitazone (maleate) |
| 162 | HY-N0529 | Rosmarinic acid |
| 163 | HY-N0148 | Rutin |
| 164 | HY-50858 | Ruxolitinib |
| 165 | HY-B1118 | Secnidazole |

| | | |
|---|---|---|
| 166 | HY-B0736A | Sertaconazole (nitrate) |
| 167 | HY-14616 | Shogaol |
| 168 | HY-13749 | Sitagliptin |
| 169 | HY-B0544 | Sodium Picosulfate |
| 170 | HY-B0334 | Sulbactam |
| 171 | HY-B1387 | Sulfamethoxypyridazine |
| 172 | HY-B2115 | Sulfogaiacol |
| 173 | HY-B0008 | Sulindac |
| 174 | HY-90009A | Tadalafil |
| 175 | HY-15295 | TAK-438 |
| 176 | HY-B0661 | Tamsulosin |
| 177 | HY-17400 | Tegafur |
| 178 | HY-13910 | Tenofovir |
| 179 | HY-B0371A | Terazosin (hydrochloride dihydrate) |
| 180 | HY-B1194 | Tetramisole hydrochloride |
| 181 | HY-B0153A | Ticlopidine (hydrochloric |
| 182 | HY-B0117 | Tigecycline |
| 183 | HY-17360 | Tiotropium Bromide |
| 184 | HY-17369 | Tirofiban (hydrochloride monohydrate) |
| 185 | HY-B0441 | Tobramycin |
| 186 | HY-B1489 | Tolmetin sodium dihydrate |
| 187 | HY-N2149 | Tomatidine |
| 188 | HY-B1496 | Tranylcypromine (hemisulfate) |
| 189 | HY-B0478 | Trazodone (hydrochloride) |
| 190 | HY-16503 | Treosulfan |
| 191 | HY-B0532A | Trifluoperazine dihydrochloride |
| 192 | HY-A0061 | Trifluridine |
| 193 | HY-B0321 | Tropicamide |
| 194 | HY-A0064 | Verapamil (hydrochloride) |

| | | |
|---|---|---|
| 195 | HY-15399 | Vigabatrin |
| 196 | HY-12053A | Vinorelbine (ditartrate) |
| 197 | HY-10440 | Vismodegib |
| 198 | HY-15414 | Vortioxetine |
| 199 | HY-B0619 | Zaltoprofen |
| 200 | HY-1321 | Zanamivir |
| 201 | HY-17413 | Zidovudine |

**Table S2. Pharmacokinetic parameters of CQD after a single dose in C57BL/6J mice.**

| Administration Route | Dose (mg/kg) | $C_{max}$ (ng/mL) | $T_{1/2}$ (h) | $T_{max}$ (h) | $AUC_{(0-\infty)}$ (h*ng/mL) | $MRT_{(0-\infty)}$ (h) |
|---|---|---|---|---|---|---|
| i.p. | 8 | 10.83±0.87 | 1.12±0.05 | 0.25±0.00 | 10.25±1.05 | 1.29±0.03 |

Data is expressed as mean ± SEM (n=3). Abbreviations: i.p. intraperitoneal injection; $C_{max}$, C peak plasma concentration of a drug after administration; $T_{1/2}$, elimination half-life; $T_{max}$, time to reach $C_{max}$; AUC, area under the concentration-time curve; MRT, mean residence time.

**Table S3. The list of 78 potential target proteins of chlorquinaldol.**

| No. | Uniprot ID | Gene Symbol | CQD/ Control Ratio | Unique Peptides | up/ down |
|---|---|---|---|---|---|
| 1 | Q8C1A3 | Mtrr | 8.408005342 | 1 | up |
| 2 | Q6P549 | Inppl1 | 5.147540792 | 4 | up |
| 3 | Q91W96 | Anapc4 | 5.031155046 | 2 | up |
| 4 | Q8K2T1 | Nmral1 | 4.815265972 | 4 | up |
| 5 | Q78JE5 | Fbxo22 | 4.373762197 | 1 | up |
| 6 | Q9D9V3 | Echdc1 | 4.373762197 | 1 | up |

| | | | | | |
|---|---|---|---|---|---|
| 7 | Q62086 | Pon2 | 3.97737505 | 1 | up |
| 8 | Q99L04 | Dhrs1 | 3.605828929 | 2 | up |
| 9 | Q5ND52 | Mrm3 | 3.38631375 | 1 | up |
| 10 | P24452 | Capg | 1.746076281 | 4 | up |
| 11 | Q6ZQL4 | Wdr43 | 1.585675855 | 1 | up |
| 12 | Q9ESD7 | Dysf | 1.504153409 | 3 | up |
| 13 | Q8VHY0 | Cspg4 | 1.42263515 | 9 | up |
| 14 | Q9CZD3 | Gars1 | 1.334687678 | 19 | up |
| 15 | Q8VHK9 | Dhx36 | 1.329254089 | 4 | up |
| 16 | Q7TMR0 | Prcp | 1.326663187 | 6 | up |
| 17 | P60670 | Nploc4 | 1.26166437 | 6 | up |
| 18 | Q8VHI3 | Pofut2 | 1.239786535 | 3 | up |
| 19 | Q60676 | Ppp5c | 0.830013921 | 1 | down |
| 20 | P62984 | Uba52 | 0.826978751 | 3 | down |
| 21 | Q60875 | Arhgef2 | 0.817908968 | 2 | down |
| 22 | Q06138 | Cab39 | 0.805942323 | 8 | down |
| 23 | Q99LC5 | Etfa | 0.802742336 | 2 | down |
| 24 | P05202 | Got2 | 0.799866424 | 18 | down |
| 25 | Q922J3 | Clip1 | 0.795153113 | 1 | down |
| 26 | P80318 | Cct3 | 0.793211512 | 20 | down |
| 27 | Q9D0F9 | Pgm1 | 0.786696065 | 15 | down |
| 28 | P62137 | Ppp1ca | 0.784266057 | 3 | down |
| 29 | Q3V1L4 | Nt5c2 | 0.77496154 | 2 | down |
| 30 | Q61205 | Pafah1b3 | 0.772218529 | 6 | down |
| 31 | Q9DC50 | Crot | 0.767099906 | 3 | down |
| 32 | Q9DBR0 | Akap8 | 0.763128399 | 1 | down |
| 33 | P59328 | Wdhd1 | 0.750943991 | 3 | down |
| 34 | Q6P1B1 | Xpnpep1 | 0.74896437 | 10 | down |
| 35 | P62715 | Ppp2cb | 0.748113937 | 1 | down |

| | | | | | |
|---|---|---|---|---|---|
| 36 | P61202 | Cops2 | 0.746301116 | 7 | down |
| 37 | Q9ERI6 | Rdh14 | 0.741532825 | 1 | down |
| 38 | Q924Y0 | Bbox1 | 0.737900939 | 1 | down |
| 39 | Q8K2T8 | Paf1 | 0.721634841 | 1 | down |
| 40 | O08529 | Capn2 | 0.720931518 | 8 | down |
| 41 | P49312 | Hnrnpa1 | 0.708057947 | 1 | down |
| 42 | Q6PEB6 | Mob4 | 0.700891709 | 1 | down |
| 43 | Q6P5F9 | Xpo1 | 0.676323848 | 5 | down |
| 44 | Q62318 | Trim28 | 0.66927705 | 6 | down |
| 45 | Q9D6N1 | Ca13 | 0.653830867 | 4 | down |
| 46 | Q9D5V5 | Cul5 | 0.650010003 | 4 | down |
| 47 | Q8R059 | Gale | 0.638698274 | 2 | down |
| 48 | P62702 | Rps4x | 0.634465108 | 11 | down |
| 49 | P46978 | Stt3a | 0.632436721 | 4 | down |
| 50 | P80314 | Cct2 | 0.623480847 | 18 | down |
| 51 | Q8K4Z3 | Naxe | 0.618061588 | 1 | down |
| 52 | Q99JF8 | Psip1 | 0.613841039 | 1 | down |
| 53 | P10649 | Gstm1 | 0.58921526 | 6 | down |
| 54 | Q3UM45 | Ppp1r7 | 0.586833015 | 7 | down |
| 55 | Q9Z1K5 | Arih1 | 0.585641521 | 2 | down |
| 56 | Q60668 | Hnrnpd | 0.558176424 | 2 | down |
| 57 | Q8BV13 | Cops7b | 0.537030935 | 2 | down |
| 58 | O70252 | Hmox2 | 0.512712757 | 1 | down |
| 59 | P18155 | Mthfd2 | 0.492938675 | 2 | down |
| 60 | P24547 | Impdh2 | 0.484484729 | 9 | down |
| 61 | Q9R1C7 | Prpf40a | 0.472290384 | 3 | down |
| 62 | P08074 | Cbr2 | 0.459377601 | 3 | down |
| 63 | Q99NH0 | Ankrd17 | 0.442379195 | 2 | down |
| 64 | O55222 | Ilk | 0.433811368 | 1 | down |

| | | | | | |
|---|---|---|---|---|---|
| 65 | Q60865 | Caprin1 | 0.424897497 | 4 | down |
| 66 | Q62348 | Tsn | 0.384832451 | 6 | down |
| 67 | Q60715 | P4ha1 | 0.375089464 | 2 | down |
| 68 | P01831 | Thy1 | 0.353934232 | 2 | down |
| 69 | Q8BMA6 | Srp68 | 0.347861767 | 4 | down |
| 70 | Q6P9P6 | Kif11 | 0.33326842 | 1 | down |
| 71 | Q8R0G9 | Nup133 | 0.17449125 | 2 | down |
| 72 | P63073 | Eif4e | 0.159210965 | 3 | down |
| 73 | O70310 | Nmt1 | 0.136543318 | 1 | down |
| 74 | Q8BXK8 | Agap1 | 0.083197327 | 2 | down |
| 75 | Q8R2Q4 | Gfm2 | 0.059213413 | 1 | down |
| 76 | P97494 | Gclc | 0.038412188 | 1 | down |
| 77 | O88874 | Ccnk | 0.017089211 | 1 | down |
| 78 | Q9EPK6 | Sil1 | 0.014062376 | 1 | down |

**Table S4. PCR primer sequences.**

| Primer | Sequence (5′- 3′) |
|---|---|
| Mouse *Mtrr*- Forward | CTGTGTCACACGGGTTTTCC |
| Mouse *Mtrr*- Reverse | TGGGGAGTGTCTTGTTGTGTATT |
| Mouse *β-actin*- Forward | GTGACGTTGACATCCGTAAAGA |
| Mouse *β-actin* - Reverse | GCCGGACTCATCGTACTCC |

**Table S5. ShRNA target sequences.**

| Gene Name | Target Sequence (5′- 3′) |
|---|---|

| | |
|---|---|
| Mouse sh*Mtrr*-1 | GCTGGTGTTGACAAACTAGAA |
| Mouse sh*Mtrr*-2 | CGTGTTTAACATCGTGGAGTT |
| Mouse sh*Mtrr*-3 | CGCTGATTACAACCGCTTCAT |
| Scramble | TTCTCCGAACGTGTCACG |
